# Cancer incidence trends in New York State and associations with common population-level exposures 2010–2018: an ecological study

**DOI:** 10.1038/s41598-024-56634-w

**Published:** 2024-03-26

**Authors:** Haokun Yuan, Rebecca D. Kehm, Josephine M. Daaboul, Susan E. Lloyd, Jasmine A. McDonald, Lina Mu, Parisa Tehranifar, Kai Zhang, Mary Beth Terry, Wan Yang

**Affiliations:** 1 Department of Epidemiology, Mailman School of Public Health, Columbia University, 722 West 168th Street, Room 514, New York, NY 10032 USA; 2 Herbert Irving Comprehensive Cancer Center, Columbia University Medical Center, New York, NY USA; 3 Department of Epidemiology and Environmental Health, School of Public Health and Health Professions, The State University of New York at Buffalo, Buffalo, NY USA; 4 Department of Environmental Health Sciences, School of Public Health, State University of New York at Albany, Rensselaer, NY USA

**Keywords:** Cancer, Environmental exposures, Lifestyle factors, Spatial patterns, Young adults, Early onset, Cancer, Environmental impact

## Abstract

The impact of common environmental exposures in combinations with socioeconomic and lifestyle factors on cancer development, particularly for young adults, remains understudied. Here, we leveraged environmental and cancer incidence data collected in New York State at the county level to examine the association between 31 exposures and 10 common cancers (i.e., lung and bronchus, thyroid, colorectal, kidney and renal pelvis, melanoma, non-Hodgkin lymphoma, and leukemia for both sexes; corpus uteri and female breast cancer; prostate cancer), for three age groups (25–49, 50–69, and 70–84 year-olds). For each cancer, we stratified by age group and sex, and applied regression models to examine the associations with multiple exposures simultaneously. The models included 642,013 incident cancer cases during 2010–2018 and found risk factors consistent with previous reports (e.g., smoking and physical inactivity). Models also found positive associations between ambient air pollutants (ozone and PM_2.5_) and prostate cancer, female breast cancer, and melanoma of the skin across multiple population strata. Additionally, the models were able to better explain the variation in cancer incidence data among 25–49 year-olds than the two older age groups. These findings support the impact of common environmental exposures on cancer development, particularly for younger age groups.

## Introduction

Cancer is a leading cause of morbidity and mortality in the United States (U.S.)^1,2^. While intrinsic factors such as sporadic mutations driven by endogenous aging processes and germline susceptibility from inherited risk variants contribute to cancer development, exogenous factors (e.g., environmental exposures and lifestyle factors), likely in combination with susceptibility, are responsible for a much larger portion of cancer risk (as much as 70–90%^3^). As such, exogenous factors can substantially affect trends in cancer^4,5^. In particular, in the U.S., cancer incidence among adults under age 40 or 50 has increased in recent years^4–7^. Identifying modifiable exogenous factors underlying these increases could thus inform early-onset cancer prevention.

Environmental exposures, at higher levels, can increase cancer risk as demonstrated in occupational cohorts (e.g., exposure to smoke and lung cancer among firefighters, and exposure to paints and lung and bladder cancers among painters^8–11^) and laboratory studies^12,13^. Environmental carcinogens can also exist at low levels in the air and water, potentially contributing to cancer risk^14–16^. Indeed, several studies have examined and found positive associations between air pollutants [e.g., particulate matters less than 10 or 2.5 μm in diameter (PM_10_ or PM_2.5_, respectively), nitrogen dioxide (NO_2_), and ozone] and certain cancers (e.g., lung, breast, and prostate)^17–24^. A more limited number of studies have examined the associations with water contaminants but found mixed results, e.g., for total trihalomethanes (TTHM)^25,26^. Nonetheless, the impact of persistent, low-dose long-term environmental exposures on cancer risk remains understudied.

Smoking is another major cancer risk factor^27^, and the reduction of smoking via regulation and behavior change has substantially reduced cancer incidence during the last few decades^28–30^. Other lifestyle factors such as obesity and physical inactivity, however, have become important contributors to cancer risk in recent years. For instance, a 2019 study found that incidence significantly increased for six obesity-related cancers in adults aged 25–49 years and more so for younger generations born since around 1950^4^. However, the relative contributions of different exogenous factors to cancer risk remain unclear. For each exogenous factor, it is also unclear if the relative risk contribution varies by cancer type or other factors such as age at diagnosis or sex.

In New York State (NYS), summary statistics indicate that statewide cancer incidence rates have been substantially above the national average^31,32^, and that like many other places in the U.S., incidence rates among young adults in NYS have increased in recent years^33^. In this work, we thus focused on examining cancer incidence trends in NYS and associations with common population-level exposures. We first compared cancer incidence rates for the most prevalent cancers in NYS with the corresponding rates nationally, among all ages and among young adults aged 25–49 years, separately. Considering the overall prevalence and prevalence among young adults, we identified 10 common cancers (see details and a full list in Sect. "Methods" and Table 1) for further analysis. Given the reported nationwide increases in early-onset cancers, we examined the changes in cancer incidence rates among young adults aged 25 – 49 years in NYS. In addition, we leveraged environmental and cancer incidence data collected in NYS at the county level to examine the association between 31 exposures and those 10 cancers, for three age groups (25–49, 50–69, and 70–84 year-olds), separately. For each cancer, stratified by age group and sex, we applied regression models to simultaneously estimate the associations with multiple exposures including environmental, social, and lifestyle factors. Our approach aims to inform county-level policies.

## Methods

**Figure 1:**
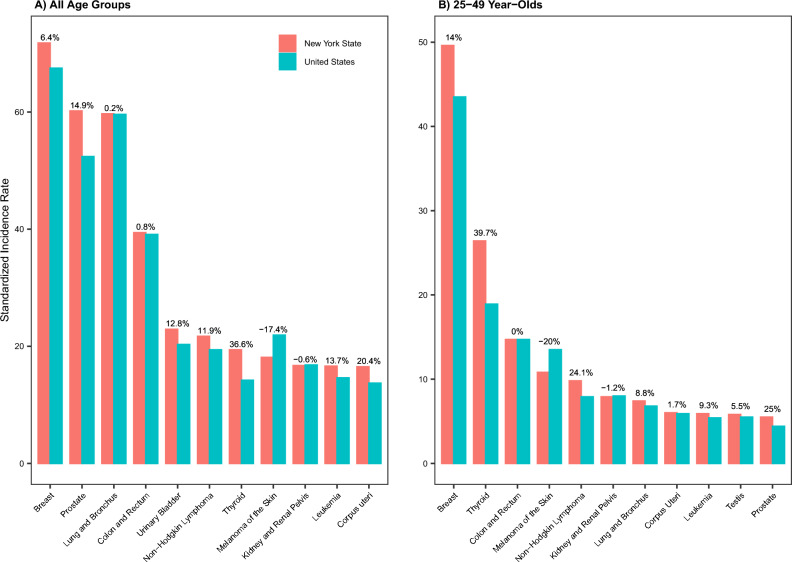
Site-specific cancer incidence rates during 2010–2018 in New York State (NYS), compared to the United States (U.S.). Bar plots show age-standardized incidence rates for the 10 most prevalent cancer types in NYS, among all ages (**A**) and 25–49 year-olds (**B**), separately. Percentages next to the bars show the percentage difference compared to the U.S., computed as (incidence rate in NYS − incidence rate in the U.S.)/(incidence rate in the U.S.) × 100%; thus, positive percentages indicate higher incidence rates in NYS than the U.S. Note that 11 cancers are shown in each panel for comparison, because the 10 most prevalent cancers among all ages and among 25–49 years were not identical in NYS during 2010–2018; cancers of the prostate and urinary bladder were among the 10 most prevalent cancers for the whole population but not for 25–49 year-olds, while cancers of the corpus uteri and testis were among the 10 most prevalent cancers for 25–49 year-olds but not for the whole population.

While cancer incidence data for NYS and most exposure data are available from the year 2000 onwards (see data availability in supplemental text and specific time periods used in Table S1), to account for a lag of approximately 10 years for cancer induction, we used cancer incidence data during 2010–2018 and risk factor data during 2000–2009 to account for an induction time of roughly 10 years. Further, we focused on cancers with the highest incidence rates for men and women in NYS, considering the prevalence both among all ages and among young adults aged 25–49 years. During 2010–2018, in NYS the 10 most prevalent cancers among all ages and among 25–49 years largely overlapped, except for cancers of the prostate, urinary bladder, corpus uteri, and testis (cancers of the prostate and urinary bladder were among the 10 most prevalent cancers for the whole population but not for 25–49 year-olds, while cancers of the corpus uteri and testis were among the 10 most prevalent cancers for 25–49 year-olds but not for the whole population). Considering the relative prevalence of these cancers (Fig. 1), we included prostate cancer and corpus uterine cancer in our time trend analysis and risk factor statistical analyses but not urinary bladder cancer or testicular cancer. In total, we included 10 cancers for men and women, separately (i.e., lung and bronchus, thyroid, colorectal, kidney and renal pelvis, melanoma of the skin, non-Hodgkin lymphoma, and leukemia for both sexes; corpus uteri and breast cancer in women; and prostate cancer in men; Table 1).

### Data sources

The cancer incidence data were obtained from the Surveillance, Epidemiology, and End Result (SEER) program (data released in April 2022)^34^. For each cancer, we calculated the age-standardized incidence rate using SEER software^35^ during the study period (2010–2018) for each age group (i.e., 25–49, 50–69, and 70–84 year-olds) and sex in each of the 62 counties in NYS. Of note, we analyzed the data by age group and sex at the county level as it is the most granular geo-unit available in the SEER program at time of this study. Incidence rates were standardized to the 2000 U.S. standard population (*n.b.,* the standardization here is to facilitate comparison across locations; as such, the specific standard population used will not affect model findings). In addition, we computed the incidence rates in NYS statewide and nationally for each of the 10 aforementioned cancer types, by age group. For the trend analysis (see below), we computed NYS statewide, site- and sex-specific annual incidence rates for each year from 2000 to 2018; note that we included the earlier years (i.e., 2000–2009) to strengthen the trend analysis.

Data for the risk factors were compiled from multiple sources (Table S1 and Supplemental Text). In brief, we included six types of measures, all at the county level:

1. Race composition and socioeconomic status (race & SES) measures based on well-documented differences in cancer incidence by race/ethnicity and SES^36,37^ (3 measures in total): the percentage of white residents, percentage of the population living in poverty, and percentage of the population without health insurance.
2. Environmental exposure measures based on evidence as reviewed in the Introduction^14–23,25,26^ (15 in total, see Table S1), including air pollutants (e.g., Ozone, NO_2_, and specific PM_2.5_ components), disinfection byproducts in drinking water (e.g., TTHM), and radon exposure.
3. General health conditions (2 measures: the prevalence of mental health problems^38,39^ and tooth loss^40–42^) and use of preventive and screening healthcare (e.g., screening for breast cancer; 5 measures in total), which may affect cancer risk and/or detection.
4. Lifestyle factors based on evidence as reviewed in the Introduction^4,27,43^ (4 measures in total): the prevalence of smoking, binge drinking, obesity, and physical inactivity.
5. Community physical characteristics that may affect or serve as proxy measures of cancer risk (2 measures: percentage of land used for agriculture and urbanization level).
6. Spatial differences based on the latitude of each county to account for potential spatial patterns (see detail in Supplemental text).

### Time trend analysis

To examine changes in early-onset cancers during 2000–2018, we analyzed the time trends in incidence among 25–49 year-olds in NYS, for the 10 aforementioned cancer types. We performed the analysis using segment log-linear regression with the joinpoint software (version 4.9.1.0; released April 2022) from the National Cancer Institute. The joinpoint models used year of diagnosis as the independent variable and log-transformed age-standardized annual cancer incidence rate as the outcome variable. The joinpoint software optimized the number of joinponts (i.e., time points when the trend changes; 0 to 2 joinpoints allowed) based on the data, and estimated the annual percent change (APC) and 95% confidence interval (CI) for each time segment as well as the average APC (AAPC) and 95% CI over the entire study period. We performed this analysis for each cancer type and sex, separately. Incidence rates were considered to increase or decrease if *P* < 0.05 from a two-sided t-distribution test; otherwise, rates were considered stable between 2000 and 2018.

### Statistical analyses to examine risk associations with environmental exposures

To examine the environmental risk factors, we first examined the goodness of fit for different model types including linear models, spatial models, Poisson models, and negative binomial models. The preliminary results indicated that these models did not outperform linear models (e.g., based on likelihood ratio test). As such, for simplicity, here we used linear regression models. Given the large number of studied risk factors (31 in total) and small number of counties (62 or less depending on the availability of data), we first used bivariable and race & SES-adjusted models to examine individual risk factors and identify the ones likely showing association with cancer risk; this subset was then further examined in a full multivariable analysis (see Fig S1 for an analysis flow chart).

All models adjusted for the three aforementioned race & SES variables (unless noted otherwise). This modeling choice was based on reported differences in cancer incidence by race/ethnicity and SES^36,37^ and our preliminary results indicating these variables affect incidence rates for most cancers studied here. For all analyses, we scaled all variables to have a mean of 0 and standard deviation of 1 to allow for comparison of the magnitude of association estimates across risk factors, as the parameters of standardized coefficients are unitless and equivalent to adjusted correlation coefficients. In addition, to ensure the robustness of model estimation, we only analyzed cancers for which > 60% of counties (i.e., 38 counties of the 62 total) reported > 5 cases over the 9-year study period for a given age group and sex.

#### Analysis of individual risk factors

We analyzed each risk factor using two simple linear models. The first model took the form of

1 $$Y_{cancer,age group, sex,T} \sim X_{r,T - 10}$$

where ${Y}_{cancer,age group, sex,T}$ is the standardized cancer incidence rate for a given age group (i.e., 25–49, 50–69, or 70–84 year-olds) and sex (men or women) during the study period *T* (i.e., 2010–2018); ${X}_{r,T-10}$ is the measure of a risk factor roughly 10 years ago, assuming a roughly 10-year lag from exposure to cancer diagnosis (see Table S1 for the exact time period for each variable and Fig S2 for a full list of variables examined for each cancer).

The second model adjusted for the three race & SES variables measured during the study period *T* (i.e., without a time-lag; ${X}_{race\&SES,T}$), per:

2 $$Y_{cancer,age group, sex,T} \sim X_{r,T - 10} + X_{race\& SES,T}$$

For most cancer types and population strata defined by age group and sex, ${X}_{race\&SES,T}$ included all three race & SES variables. However, for a few instances, the percentage of white residents was highly correlated with certain risk factors (e.g., smoking prevalence among 25–49 year-old women) and thus not included. See Fig S2 for detail and sensitive analyses below.

Risk factors with a *P*-value < 0.1 from either of the two models (i.e., Eqs. 1 and 2) were then pooled (see Fig S2 for specific risk factors selected) and examined further in the full multivariable analysis.

#### Multivariable analysis

In this analysis, for each cancer, age group, and sex, we aimed to identify the best-fit model selected from the pooled risk factor subset from the individual risk factor analysis and two spatial covariates. The two spatial variables (i.e., each county’s latitude and a polynomial term of latitude to capture potential nonlinear effect) were included here to account for and identify potential spatial patterns per model goodness-of-fit. In addition, there are two challenges. First, it is computationally expensive to test all combinations of variables (e.g., there are > 2 × 10^9^ combinations for 31 variables). Second, certain variables are highly correlated and should not be included in the same model due to multicollinearity^44^ (e.g., the different components of PM_2.5_; see Figs S3-4 for the pairwise correlations of all variables). Thus, we first computed the pairwise Pearson’s correlation (*r*) and listed all compatible combinations such that no risk factor pairs with an *r* > 0.6 were included in the same combination (see Sensitivity analyses below). This “decorrelation” step breaks the risk factor pool into smaller subsets to mitigate both the computational challenge and multicollinearity issue. Of note, because we aimed to identify key environmental cancer risk factors here, we did not use regularization approaches (e.g., the LASSO)^45^ due to the inconsistency, particularly for small datasets, in variable selection through cross-validation.

For each “decorrelated” risk factor combination (${X}_{comb, T-10}$), we use the R package “leaps”^46^ to perform an exhaustive search for the best subset of the variables in ${X}_{comb,T-10}$ that best fitted the cancer data (here, the one with the lowest Bayesian information criterion (BIC)^47^). For a cancer-age group-sex dataset with *N* ${X}_{comb, T-10}$ subsets, we thus obtained *N* best models. To select the final best model, we pooled the *N* models, excluding those with an adjusted R^2^ < 0.3 to ensure that all remaining models can explain at least 30% of the variation in the cancer incidence data. After removing duplicates (as the same model could be selected from different ${X}_{comb, T-10}$ subsets), nested models (i.e., models sharing the same subset of covariates) could still exist, because they were selected independently from different ${X}_{comb, T-10}$ subsets. As such, if a group of nested models largely outperforms other groups, multiple nested models would occupy the top ranks even though they do not provide much additional information on risk factors. To address this issue, we further identified all nested models within the pool and only retained the best-fit model with the lowest BIC for each group of nested models. We then ranked these unique top models and deemed the one with the lowest BIC as the final best-fit model (Fig S1). However, in the event that there were multiple models with similar BICs (e.g., the difference in BIC is < 2), we deemed those models comparable and presented all of them for discussion. Taken together, the final model took the following general form:

3 $$Y_{cancer,age group, sex,T} \sim X_{best.subset,T - 10} + X_{race\& SES,T}$$

Table 1 shows the specific ${X}_{best.subset,T-10}$ and ${X}_{race\&SES,T}$ for each cancer, age group, and sex.

#### Sensitivity analyses

We conducted three sets of sensitivity analyses. First, we relaxed the inclusion criterion of *P* < 0.1 (main analysis in the individual risk factor analysis), to *P* < 0.2 or *P* < 0.3; this would allow more variables to be examined in the subsequent multivariable analysis. Second, in the multivariable analysis, we reduced the correlation threshold controlling covariate collinearity from *r* < 0.6 (main analysis) to *r* < 0.5; this would allow fewer corelated variables to be in the same model. Third, to test variables highly correlated with race (i.e., percentage of white residents, one of ${X}_{race\&SES,T}$ variables), we did not adjust for race as in the main analysis; instead, race was treated as a potential covariate and selected based on BIC along with other variables.

All model analyses were conducted using R language (version 4.0.2^48^). We report the mean and 95% CI for each association estimate and statistical significance at *a* = 0.05 level. Here we did not adjust the *P*-values for multiple comparisons, due to the small number of covariates included in all best-performing models (≤ 7 covariates; Table 1); in addition, not applying the adjustment would help reduce type II error for associations that are not null^49^.

## Results

### Cancer incidence rates in NYS compared to the national rates

Figure 1 shows a comparison of incidence rates during 2010–2018 in NYS and nationally. Combining all ages, NYS shared 9 of the 10 most prevalent cancers with the U.S. (leukemia was the 10th most common cancer in NYS, while cervical cancer was the 10th most common cancer in the U.S.). For most of these cancers (Fig. 1A), incidence rates were higher in NYS than the U.S. overall, ranging from 0.2% higher for lung and bronchus cancer to 36.6% higher for thyroid cancer (Fig. 1A). Among young adults aged 25–49 years, incidence rates of several cancers were also higher in NYS than the U.S. (in particular, by 14% for breast cancer, by 25% for prostate cancer, by 39.7% for thyroid cancer, and by 24.1% for non-Hodgkin lymphoma; Fig. 1B).

### Trends in cancer incidence among young adults (25–49 year-olds) in NYS

**Figure 2:**
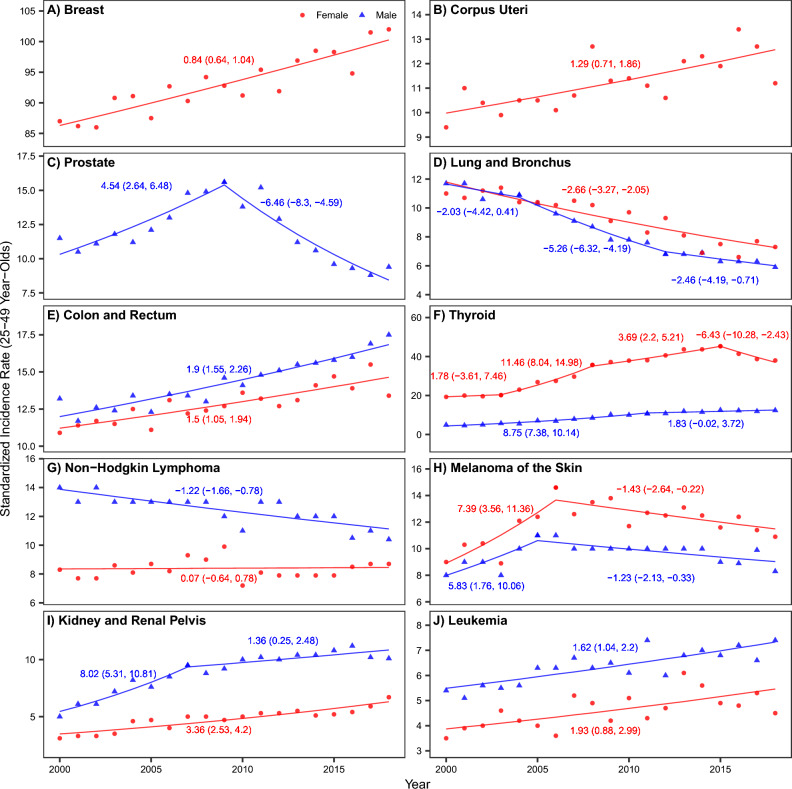
Trends in cancer incidence among young adults (25–49 year-olds) in New York State (**A**–**J**) for each of the 10 common cancers). Dots (red • = women and blue ▲ = men) show annual cancer incidence rates for each cancer type (see subplot title). Line segments show time periods with different time trends, as identified by the joinpoint software; numbers next to each line segment show estimated annual percent changes (and 95% confidence intervals) for each time period (red = women and blue = men).

As noted in the Introduction, recent studies have reported increases in early-onset cancers in the U.S.^4–7^. To examine whether there have been similar increases in early-onset cancers in NYS, we examined the changes in incidence for 10 common cancers among 25 – 49 year-olds. Using joinpoint trend analysis, we estimated that six cancer types significantly increased in incidence during 2000–2018 (Fig. 2). This included female breast cancer [AAPC: 0.84% (95% CI 0.64–1.04%)], cancer of the corpus uteri [AAPC: 1.29% (95% CI 0.71–1.86%)], colorectal cancer [AAPC: 1.9% (95% CI 1.55–2.26%) for men, and 1.5% (95% CI 1.05–1.94%) for women], thyroid cancer [AAPC: 6.0% (95% CI 4.99–7.03%) for men, and 3.67% (95% CI 2.33–5.04%) for women], cancer of the kidney and renal pelvis [AAPC: 3.9% (95% CI 2.77–5.04%) for men, and 3.36 (2.53–4.2%) for women], and leukemia [AAPC: 1.62% (95% CI 1.04–2.2%) for men, and 1.93 (0.88–2.99%) for women], during 2000–2018.

### Associations between county-level environmental factors and cancer incidence rates

Table 1 summarizes the incidence rates for the 10 types of cancer (8 for men and 9 for women) in NYS by age group and sex, and the best-fit models for each cancer and population stratum defined by age and sex. In total, 642,013 incident cancer cases (304,916 among men and 337,097 among women) were included in this study. The models were able to explain at least 30% of the variation in incidence data for six cancers among 25–49 year-olds (i.e., lung and bronchus, melanoma of the skin, thyroid in both men and women; kidney and renal pelvis, breast, and corpus uteri in women; Table S2), five cancers among 50–69 year-olds (i.e., lung and bronchus, melanoma of the skin, thyroid in both men and women; female breast; and prostate; Table S3), and four cancers among 70–84 year-olds (i.e., lung and bronchus in both men and women; melanoma of the skin in men; thyroid in women; and prostate; Table S4). Tables S2–S4 show specific association estimates for each age group. Results from the three sets of sensitivity analyses are in general consistent with those from the main analysis (see Tables S5–S7 and Supplemental Text). Below we focus on summarizing the identified common risk factors across multiple population strata.

**Table 1:** Summary of the 10 cancers examined and risk factors identified in the best-fit models.

Sex	Age	Cancer	No. counties included	Cumulative incident case count	Standardized incidence rate	Risk factors*	Adjusted R^2^
Men	25–49	Colon and rectum	61	4444	16.7 (6)	POV, INS, RACE, RADONZONE	0.07
		**Melanoma of the skin**	**58**	**2687**	**12.7 (4.6)**	**POV, INS, RACE, TOXIC, NIT**	**0.54**
		Kidney and renal pelvis	51	2915	11.1 (4.2)	POV, INS, RACE, PHYACT	0.12
		Non-Hodgkin lymphoma	51	3288	9.9 (3.3)	POV, INS, RACE, AG	0.15
		**Thyroid**	**47**	**3282**	**9.8 (3.9)**	**POV, INS, RACE, PHYACT, BC**	**0.30**
		Prostate	48	3300	8.8 (4.4)	POV, INS, RACE, OZONE	0.23
		**Lung and bronchus**	**49**	**1941**	**8.4 (3.4)**	**POV, INS, RACE, SMOK, TTHM, LAT**	**0.57**
		Leukemia	48	1951	7.9 (5.6)	POV, INS, RACE, RURAL	0.24
	50–69	**Prostate**	**62**	**81,193**	**350 (62)**	**POV, INS, RACE, OZONE, OBESE**	**0.32**
		**Lung and bronchus**	**62**	**27,331**	**155.2 (30.2)**	**POV, INS, RACE, SMOK, LAT**	**0.72**
		Colon and rectum	62	18,867	89.1 (10.1)	POV, INS, RACE, PHYACT	0.09
		Kidney and renal pelvis	61	11,739	56.3 (11)	POV, INS, RACE, LAT, LAT2	0.16
		**Melanoma of the skin**	**62**	**8950**	**50.4 (14.5)**	**POV, INS, RACE, RURAL, PHYACT, OBESE, SOIL**	**0.51**
		Non-Hodgkin lymphoma	61	10,112	46.8 (7.3)	POV, INS, RACE, NITRA	0.15
		Leukemia	61	7425	37 (7.7)	POV, INS, RACE, OMOC	0.10
		**Thyroid**	**55**	**4,169**	**16.8 (5.7)**	**POV, INS, RACE, LAT, LAT2**	**0.48**
	70–84	**Prostate**	**62**	**41,971**	**644.7 (113.2)**	**POV, INS, RACE, OZONE, LAT, NIT**	**0.38**
		**Lung and bronchus**	**62**	**26,775**	**507.8 (79.8)**	**POV, INS, RACE, AG, SMOK, LAT**	**0.54**
		Colon and rectum	61	13,846	230.1 (44.3)	POV, INS, RACE, RURAL, UTDPRV1	0.23
		**Melanoma of the skin**	**61**	**7207**	**136.9 (39.7)**	**POV, INS, RACE, TOXIC, UTDPRV1, SOIL**	**0.39**
		Non-Hodgkin lymphoma	61	7622	128.9 (24.2)	POV, INS, RACE, OM	0.13
		Leukemia	61	6561	116.3 (27.9)	POV, INS, RACE, RADONZONE	0.18
		Kidney and renal pelvis	61	6124	102.9 (19.7)	POV, INS, RACE, OBESE, OM	0.18
Women	25–49	**Breast**	**61**	**28,204**	**89.5 (14.3)**	**POV, INS, RACE, NH4**	**0.42**
		**Thyroid**	**61**	**12,111**	**37.6 (10.4)**	**POV, INS, RACE, AG, PHYACT, LAT**	**0.45**
		**Melanoma of the skin**	**60**	**3607**	**19 (7.6)**	**POV, INS, RACE, AG**	**0.53**
		Colon and rectum	57	4053	16.2 (6.6)	POV, INS, RACE, RURAL	0.13
		**Corpus uteri**	**55**	**3470**	**14.5 (6.8)**	**POV, INS, RACE, LAT**	**0.34**
		**Lung and bronchus**	**55**	**2385**	**11.6 (5.2)**	**POV, INS, RURAL, SMOK**	**0.46**
		**Non-Hodgkin lymphoma**	**39**	**2411**	**7.1 (3.3)**	**POV, INS, RACE, RURAL, PHYACT, NH4**	**0.32**
		**Kidney and renal pelvis**	**42**	**1,608**	**6.9 (3.5)**	**POV, INS, RACE, RURAL, CHKUP, AG**	**0.40**
	50–69	**Breast**	**62**	**70,389**	**303.8 (26)**	**POV, INS, RACE, MAMMO**	**0.42**
		**Lung and bronchus**	**62**	**26,582**	**142 (30.5)**	**POV, INS, RURAL, SMOK, LAT**	**0.60**
		Corpus uteri	62	21,181	94.9 (11.3)	POV, INS, RACE, NIT	0.22
		Colon and rectum	61	14,945	64.7 (11.4)	POV, INS, RACE, RURAL	0.13
		**Thyroid**	**61**	**10,735**	**39.8 (12.6)**	**POV, INS, RACE, AG, COLSIG**	**0.47**
		Non-Hodgkin lymphoma	61	8,286	35.7 (8.6)	POV, INS, RACE, LAT, LAT2	−0.05
		**Melanoma of the skin**	**61**	**6,255**	**34.3 (9.8)**	**POV, INS, RACE, AG**	**0.41**
		**Kidney and renal pelvis**	**61**	**5,679**	**28 (6.7)**	**POV, INS, RACE, RURAL, RADONZONE, COLSIG, TTHM**	**0.32**
		Leukemia	59	4,937	22.6 (6.6)	POV, INS, RACE, LAT	0.13
	70–84	Breast	62	36,218	474.4 (65.6)	POV, INS, RACE, NIT	0.20
		**Lung and bronchus**	**62**	**27,660**	**394.8 (68.5)**	**POV, INS, SMOK**	**0.42**
		Colon and rectum	62	14,032	189.7 (39)	POV, INS, RACE, MAMMO	0.13
		Corpus uteri	61	9,026	111 (21)	POV, INS, RACE, COLSIG, NH4	0.16
		Non-Hodgkin lymphoma	60	7,060	90.6 (19.8)	POV, INS, RACE, OZONE	0.12
		Leukemia	60	4,563	60.4 (15.7)	POV, INS, RACE, CHKUP	0.09
		Melanoma of the skin	60	3,950	53.7 (18.5)	POV, INS, RACE, HAA5	0.21
		Kidney and renal pelvis	57	3,771	51.9 (15.1)	POV, INS, RACE, NITRA	0.13
		**Thyroid**	**40**	**2,452**	**24.4 (9.8)**	**POV, INS, RACE, MAMMO, COLSIG, AG**	**0.35**

Columns 4 and 5 show the number of counties included and age-standardized cancer incidence rate (per 100,000 people; mean and standard deviation in parentheses), for each cancer, age group, and sex (specified in columns 1–3). Columns 6 and 7 show the risk factors identified in the best-fit model and the corresponding adjusted R^2^ (those with an adjusted R^2^ > 0.3 are bolded). See specific measure corresponding to each risk factor abbreviation in the footnote and details in Table S1. Note Leukemia for women aged 25–49 and thyroid cancer for men aged 70–84 were not included in the analysis due to low incidence rates (i.e., less than 60% of counties reported > 5 cases over the 9-year study period).

* POV: percent of population living in poverty; INS: percent of population without health insurance; RACE: percentage of white residents; RADONZONE: radon zone; TOXIC: rate of reported acute toxic substance release incidents per 100,000 population; NIT: annual mean nitrate concentration in ambient air; PHYACT: percent of adults aged >  = 18 years with no leisure-time physical activity; AG: percent of land used for agriculture; BC: Annual mean black carbon concentration in ambient air; OZONE: number of days with maximum 8-h average ozone concentration exceed NAAQS; SMOK: percent of adults aged >  = 18 years with current smoking; TTHM: mean concentration of total trihalomethanes in drinking water; LAT: latitude of county; LAT2: polynomial term of latitude; RURAL: classification of county from rural to urban; OBESE: percent of adults aged >  = 18 years with obesity; SOIL: annual mean mineral dust concentration in ambient air; NITRA: mean concentration of nitrate in drinking water; OMOC: spatially and seasonally resolved estimate of the ratio of global organic mass to organic carbon; UTDPRV: percent of older adult aged >  = 65 years who are up-to-date on a core set of clinical preventive services; OM: annual mean organic matter concentration in ambient air; NH4: annual mean ammonium concentration in ambient air; CHKUP: percent of adults aged >  = 18 years with visits to the doctor for routine checkup; MAMMO: percent of women aged >  = 50 to = 74 years who use mammography; COLSIG: percent of adults aged >  = 50 to = 75 years who have received a fecal occult blood test, sigmoidoscopy, or colonoscopy; HAA5: Mean concentration of haloacetic acids in drinking water.

#### Environmental risk factors

Table 2 summarizes all environmental exposures identified in this study, adjusting for race & SES and non-environmental exposures. Most notably, models identified several PM_2.5_-related variables to be positively associated with several cancers in men (i.e., prostate, thyroid, and melanoma of the skin; estimated mean association ranged from 0.24 to 0.69, all with *P* < 0.05; Table 2). In addition, consistent with results reported in the literature^50^, for female breast cancer cases diagnosed at age 25–49 years, the model estimated positive associations with ambient PM_2.5_ concentrations [estimated association: 0.3 (95% CI 0.06–0.53) with the ${\text{NH}}_{4}^{ + }$ component as shown in Table 2, or 0.27 (95% CI 0.05 0.49) with the ${\text{SO}}_{4}^{ + }$ component from another model with similar performance].

**Table 2:**
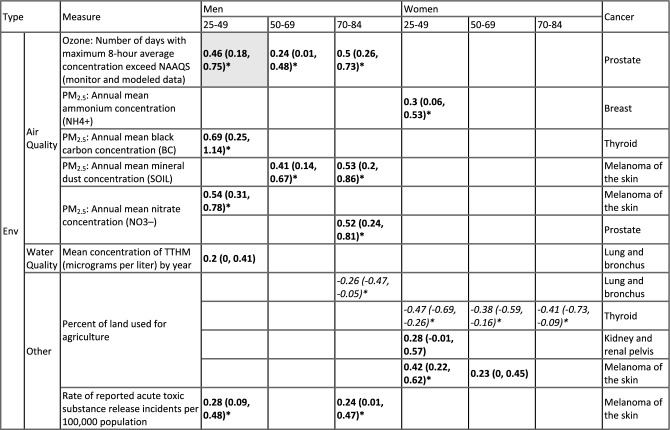
Estimated associations with environmental risk factors.

All estimates here adjusted for other variables as detailed in the main text and Table 1. First column shows the type of environmental variable; 2nd column (“Measure”) shows specific measures. The last column shows the type of cancer for which the estimates were made. The middle panel show estimated associations (see corresponding sex, and age group on the top). To allow for comparison of the magnitude of association estimates across risk factors, we standardized the variables to 0 mean and 1 standard deviation. Thus, the estimates (mean and 95% confidence intervals in parentheses) are the parameters of standardized coefficients and equivalent to adjusted correlation coefficients. Colors indicate the direction of the association (Italics = negative; Bold = positive). Asterisks (*) indicate statistically significant at a = 0.05 level. Grey cells indicate the association is from a model with an adjusted R^2^ < 0.3 (see Table 1 for detail). Blank cells (i.e., no estimates) indicate the measure was not identified in the best-fit model for the corresponding cancer type, sex, and age group, or not applicable for the sex-specific cancers (e.g., not estimates prostate cancer among women).

We further examine whether there are common environmental risk factors across population strata, for each cancer. For this purpose, estimates for all six population strata (3 age groups × 2 sexes) are examined, including one model with an adjusted R^2^ < 0.3 (Table 2). Among the fifteen environment variables examined here, for men, models estimated positive associations, consistent across age groups, between ambient ozone concentration and prostate cancer (estimated mean association ranged from 0.24 to 0.50), and between mineral dust concentration measured in ambient PM_2.5_ (estimated mean association ranged from 0.41–0.53) and acute toxic substance release incidence rate (estimated mean association ranged from 0.24–0.28) and melanoma of the skin. For women, models estimated negative associations, across all age groups, between percentage of land used for agriculture and thyroid cancer (estimated mean association ranged from − 0.47 to − 0.38; Table 2).

#### Non-environmental risk factors

Table 3 summarizes the identified common non-environmental risk factors across the six population strata, for each cancer, adjusting for race & SES and environmental exposures. Among the three race & SES variables, models estimated that counties with higher poverty prevalence and lower health-insurance coverage had lower incidence rates of breast cancer in women and thyroid cancer for both men and women (estimated mean association ranged from -0.49 to  -0.12 among 25–49 and 50–69-year-olds; Table 3); these associations are consistent with results reported in the literature (e.g.^51–53^ for breast cancer). Also consistent with the literature^54–56^, models estimated that counties with a higher proportion of white residents had higher incidence rates of melanoma of the skin for all age groups and both sexes (estimated mean association ranged from 0.23–1.07), as well as higher incidence rates of uterine cancer for all age groups (estimated mean association ranged from 0.18–0.87).

**Table 3:**
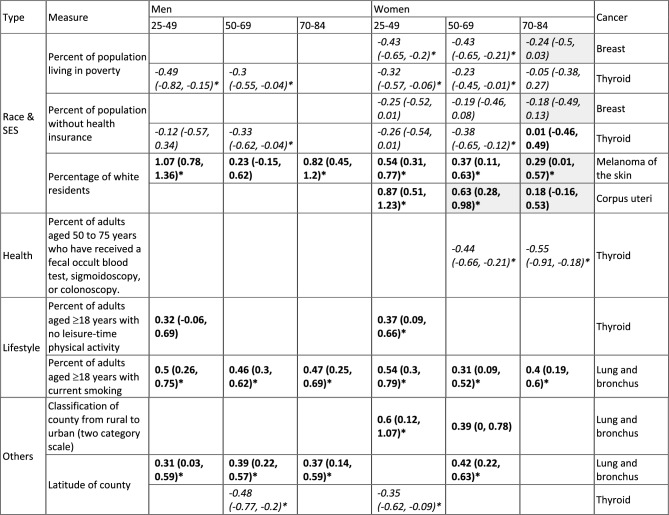
Common non-environmental risk factors.

All estimates here adjusted for other variables as detailed in the main text and Table 1. First column shows the type of variable; 2nd column (“Measure”) shows specific measures. The last column shows the type of cancer for which the estimates were made. The middle panel show estimated associations (see corresponding sex, and age group on the top). To allow for comparison of the magnitude of association estimates across risk factors, we standardized the variables to 0 mean and 1 standard deviation. Thus, the estimates (mean and 95% confidence intervals in parentheses) are the parameters of standardized coefficients and equivalent to adjusted correlation coefficients. Colors indicate the direction of the association (Italics = negative; Bold = positive). Asterisks (*) indicate statistically significant at a = 0.05 level. Grey cells indicate the association is from a model with an adjusted R^2^ < 0.3 (see Table 1 for detail). Blank cells (i.e., no estimates) indicate the measure was not identified in the best-fit model for the corresponding cancer type, sex, and age group, or not applicable for the sex-specific cancers (e.g., not estimates prostate cancer among women).

Among the seven healthcare-related variables examined here, for women, models estimated that counties with higher coverage of colorectal cancer screening had lower thyroid cancer incidence rates, for the screening-eligible age groups (estimated mean associations -0.44 and -0.55 for 50–69 and 70–84-year-olds, respectively; Table 3).

Among the four lifestyle factors examined here, models estimated positive associations between smoking and lung cancer (estimated mean association ranged from 0.31 to 0.54), for all age groups and both sexes. In addition, models estimated positive associations between physical inactivity and thyroid cancer among 25–49-year-old men and women (estimated mean associations 0.32 and 0.37, respectively; Table 3).

Lastly, models identified two likely spatial patterns. Counties in northern NYS were estimated to have higher lung cancer incidence rates for 4 of the 6 population strata (i.e., except for 50–69 and 70–84-year-old women; estimated mean associations with county latitude ranged from 0.31 to 0.42; Table 3 and Fig S5), and lower thyroid cancer incidence rates for 3 of the 6 population strata (estimated mean associations with county latitude ranged from -0.48 to -0.35; Table 3 and Fig S5).

## Discussion

Due to the low levels of exposures and long cancer induction time, it is challenging to assess the impact of common environmental, social, and lifestyle exposures on cancer development using individual-level cohort data, particularly for younger adults for whom the absolute risk is low. Here we have leveraged multiple publicly available datasets to examine the associations of 31 exposures for the 10 common cancers in NYS. Overall, we found several population-level common risk factors, including positive associations between ambient air pollutants (ozone and PM_2.5_) and prostate cancer, female breast cancer, and melanoma of the skin, positive associations between smoking and lung cancer, and positive associations between physical inactivity and thyroid cancer across multiple population strata defined by age and sex (Tables 2 and 3).

In the last few decades, incidence rates of several cancers (e.g., breast, colorectal, kidney, thyroid, lymphoma, and leukemia ^4,6,7^) among young adults have increased. Several lifestyle factors (e.g., obesity^4^) have been proposed as contributors but the underlying drivers of these cancer trends remain unclear. Analyzing incidence trends among 25–49 year-olds, we found that, like many other regions of the U.S., NYS has seen significant increases in six early-onset cancers (Fig. 2). In this study, we were unable to examine the changes in exposures over time that may contribute to the increases in early-onset cancers. Nonetheless, we leveraged the spatial differences in exposures to help identify the underlying drivers. For young adults aged 25–49, we found positive associations of ambient PM_2.5_ concentration with breast cancer in women and with thyroid cancer and melanoma of the skin in men (Table S2). These findings, consistent with the literature^50,57^, highlight the negative impact of persistent air pollution on cancer development, including for young adults. In addition, also consistent with the literature^58,59^, we found positive associations of smoking with lung cancer and physical inactivity with thyroid cancer among 25–49 year-olds for both men and women (Table S2). These findings add to the growing literature on underlying etiologic factors that may be driving the recent increases in early-onset cancers.

More generally, we found the models were able to better explain the variation in cancer incidence data among 25–49 year-olds than the two older age groups (9 models for 6 cancers in 25–49 year-old men or women vs. 8 models for 4 cancers in 50–69 year-olds and 5 models for 4 cancer in in 70–84 year-olds had an adjusted R^2^ > 0.3). The greater explanatory power for younger adults may reflect greater relative risk contributions of exogenous factors during earlier life than for older ages when intrinsic aging processes may have greater influence on cancer risk. Together with the growing evidence supporting the importance of early-life exposures^60,61^, these findings suggest policies that can reduce key exposures for young adults may prove fruitful in reversing the recent increases in early-onset cancers.

Although group-level analyses may suffer from ecological fallacy when trying to extrapolate to individual-level mechanisms, ecological studies may actually be suitable for population-level policies, particularly when considering environmental exposures and other macro-level determinants of health. Here, the strength of our study includes a robust examination of multiple types of risk factors, which helps reveal key insights to inform future studies and policy making. We comprehensively accounted for multiple types of exposures/variables, including race & SES, environmental exposures, lifestyles, healthcare access, and community physical characteristics. In addition, we included a 10-year lag to account for the time lag from exposure to cancer development.

Another strength of our study is the stratification by age group and sex. This allows the identification of risk factors for specific age group of interest (e.g., younger adults aged 25–49). It also allows comparison across multiple population strata to identify common risk factors, which can help elucidate underlying risk mechanisms (e.g., shared pathways of carcinogenesis) and inform intervention targets. For example, the models identified several components of PM_2.5_ to be positively associated with several cancers in men (Table 2), suggesting this exposure may be particularly relevant and harmful to men. As noted in previous work^62,63^, identifying the elemental components of PM_2.5_ associated with increased cancer risk may help improve the understanding of pathomechanisms and the identification of relevant sources of PM_2.5_. Previous studies have also reported positive associations between several components of PM_2.5_ and cancers^63–67^. Our findings add to this literature. In addition, the models were able to identify two likely spatial patterns for lung and thyroid cancer in NYS, which could inform policy making and public health action (e.g., resource allocation and enhanced interventions in regions with higher incidence rates).

We also recognize several study limitations. First, our study was an ecological analysis, which only provides association estimates at the population level, rather than causal inference. As reviewed above, however, this can be viewed as a strength if the group level is the unit for intervention. Second, due to a lack of available data, we were unable to examine many other environmental exposures such as personal care products and specific chemicals (e.g., polycyclic aromatic hydrocarbons (PAHs)^68,69^ and per- and polyfluoroalkyl substances (PFAS)^70^). Third, for several key cancers (e.g., colorectal cancer and leukemia, for which incidence rates have increased among young adults), the variables included here were insufficient to explain the incidence data and thus not examined further here. This is likely in part due to the limited statistical power of ecological study design and/or data limitations. In particular, there were only 62 counties in this study and county-level data may not capture individual-level heterogeneities in exposures. Similar limitations may have hindered our ability to identify weaker population-level exposures (e.g., radon as a cause of lung cancer^71^ and leukemia^72^). Nonetheless, it also highlights the challenges in identifying underlying risk factors for these cancers and the need for more in-depth investigations, given the rapid incidence increases in recent years.

The fourth limitation is that, as noted in the Sect. "Methods", to adjust for race/ethnicity and SES that may affect cancer risk and/or detection, we included three race & SES variables in the models. However, some of these race & SES variables (e.g., poverty and race) were highly correlated with the obesity data, which may have limited the models’ ability to identify obesity as a risk factor for cancers known to have a positive association (e.g., postmenopausal breast cancer and colorectal cancer; Table S8). Fifth, the time-lag of carcinogenesis could vary by cancer and risk factor; thus, the 10-year lag used here may be insufficient for cancer-risk factor pairs with a longer induction time. Relatedly, cross-county relocation could occur over a long period of time (e.g., 10 years) and affect the duration of exposure; we were unable to account for such changes due to a lack of data. Future work could examine different time-lags with more accurate exposure classification, when more detailed, longer term data become available. Lastly, our study focused on NYS; this smaller spatial scale and more homogeneous exposures across counties may have limited the models’ ability to identify other potential associations. However, as noted in the introduction, the more comprehensive data available for NYS allowed examination of a larger set of environmental exposures. In addition, with the modeling strategy developed here, future work can expand to include other states and counties across the U.S.

Despite the above limitations, our analyses do support an efficient step to gather evidence on the impact of common environmental exposures and cancer development particularly for younger age groups that are not often included in cancer cohorts. Here we show that, at a population level, we were able to consistently identify previously reported cancer risk factors (e.g., smoking and physical inactivity) as well as develop new evidence on the role of air pollution on multiple cancers. Importantly, our analyses also identify significant increases in several key cancers among young adults in NYS during recent years (particularly, cancers of the breast and corpus uteri among 25–49-year-old women; cancers of the colon and rectum, thyroid, kidney and renal pelvis, and leukemia among 25–49 year-olds for both men and women); and model results suggest there may be greater relative risk contributions of exogenous factors during earlier life than for older ages. More in-depth studies looking into the impact of key exposures and windows of susceptibility (e.g., related air population and physical inactivity during early life, as preliminarily identified here) are thus warranted. Hopefully, improved understanding will better inform policies to more effectively reduce key exposures during key susceptible windows and better prevent early-onset cancers.

### Supplementary Information

Supplementary Information.

## Data Availability

Data used in this study are publicly available on Github (https://github.com/YangLab-CU/NYS_cancer_env_exposure).
