# Supplementary material for: Cancer incidence trends in New York State and associations with common population-level exposures 2010–2018: an ecological study
Source: Sci Rep. 2024 Mar 26;14:7141. doi: 10.1038/s41598-024-56634-w (PMC10966002; doi:10.1038/s41598-024-56634-w)

**Supplemental Materials**

**for**

**Cancer incidence trends in New York State and associations with common population-level exposures 2010-2018: an ecological study**

Haokun Yuan, Rebecca D. Kehm, Josephine M. Daaboul, Susan E. Lloyd, Jasmine A. McDonald, Lina Mu, Parisa Tehranifar, Kai Zhang, Mary Beth Terry, Wan Yang

This document includes:

1. Supplemental Text, with additional details on i) data sources, specific measures used, and data processing; and ii) results of sensitivity analyses.
2. Supplemental Tables 1-8
3. Supplemental Figures 1-5

**Supplemental Text**

***Processing of risk factor data***

Table S1 summarizes the risk factors examined in this study, including specific measures used, data sources, time-period of measures used, and summary statistics for each measure. For the measurement time-period, whenever possible, we used exposure-related risk factor data during 2000 – 2009 to examine the associations with cancer incidence rates during 2010-2018, to account for the induction time (i.e., a roughly 10-year time lag) from exposure to cancer development. Below, we detail specific data availability (e.g., missing data during the 2000 – 2009 period), processing procedure (e.g., mapping, averaging, and handling of missing data), and additional considerations (e.g., choice of measures).

1) Environmental exposure risk factors

Environmental exposure risk factors assessed here include measures of ambient air quality, drinking water quality, toxic substance release, and radon level. For ambient air quality, we included both gaseous pollutants (i.e., ozone and nitrogen dioxide) and particulate matter (i.e., PM_2.5_ and organic fraction of the particles). There are multiple ozone (O_3_) concentration measures [1, 2]. Here, we used the number of days during a year with a maximum 8-hour average concentration exceeding the National Ambient Air Quality Standard (i.e., 0.075 ppm) as a proxy measure for ozone. These data were based on ozone concentrations taken by air monitors and, for locations without such ground-level measures, estimated using models by the Environmental Protection Agency (EPA). Data for years 2000 - 2009 were averaged and used in our analyses [3, 4]. Nitrogen dioxide (NO_2_) are another EPA criteria air pollutant [5]. As ground-level measurements were sparse and unavailable for most counties in NYS, here we instead used model-estimated NO_2_ concentrations generated by the Atmospheric Composition Analysis Group (ACAG) at Washington University in St. Louis [6]. These estimates were provided at ~1 km x 1 km resolution; we further mapped the estimates by geolocation and extracted the county-level estimates for NYS. As the NO_2_ estimates were not available before 2005, we averaged estimates from 2005 to 2009 for use in our analyses. For PM_2.5_, we are interested in whether any specific component of PM_2.5_ [i.e., black carbon (BC), organic matter (OM), sulfate (SO_4_), ammonium (NH_4_), nitrate (NO_3_), mineral dust (SOIL), and sea salt (SS)] has a particularly strong association with cancer incidence rate. Thus, instead of using the total PM_2.5_ concentration, we calculated the concentration of each PM_2.5_ component in the air by multiplying the annual PM_2.5_ concentration data monitored and modeled by the EPA [3] with the percentage of that PM_2.5_ component in NYS counties estimated by ACAG from 2000 to 2009 [7, 8]. The organic fraction of particles, usually measured by the ratio of organic mass to organic carbon (OMOC), is also of concern [9].To examine its association with the studied cancers, we used the estimates from ACAG (i.e., estimated for 2005-2008,[10]). Other EPA criteria air pollutants (in particularly, carbon monoxide and sulfur dioxide) were not analyzed here, due to a lack of data.

In addition to ambient air quality, we were also interested whether chemicals (especially disinfection byproducts) in drinking water are associated with cancer incidence rate. The National Environmental Public Health (EPH) tracking network reported multiple chemical concentrations in drinking water, including arsenic, nitrate, TTHM, haloacetic acids (HAA5), atrazine, di(2-ethylhexyl)phthalate (DEHP), tetrachloroethylene (PCE), trichloroethylene (TCE), and radium [11]. However, many of these chemicals (e.g., arsenic, atrazine, and DEHP) were measured using different methods in different counties (i.e., different detection limits used in different counties; and during our study period, 60%-95% of counties had values below the specific detection limits and reported as ‘Not Detected’) and thus not comparable across counties. A few other measures (i.e., PCE and TCE) did not show variations across counties (i.e., measured values were the same for all counties). Thus, here, we only included three measures (i.e., TTHM, HAA5, and NITRA) without the above issues. For these three measures, drinking water samples were collected from the community water system (CWS), and the population each CWS served varied from hundreds to millions. For a county served by multiple CWSs, we computed the average as the chemical concentrations in all CWSs in the county weighted by the percentages of CWS service population. For New York City (NYC), data of TTHM and HAA5 concentration were missing for some of its five counties. Because the five counties in NYC shared a major CWS serving over 8 million people, we imputed the missing values using the average of all available measures from NYC counties.

Other environmental risk factors included in the analysis were toxic substance release and radon concentration. Toxic substance release was defined as any chemical, biological or radiological substance that can cause harm upon exposure [12] and was measured by the rate of reported acute toxic substance release incidents per 100,000 population from 2000 to 2009 [13]. The top 10 toxic substances most frequently associated with toxic substance release incidents during 1996 ‑ 2011 were carbon monoxide, ammonia, paint, sodium hydroxide, hydrochloric acid, sulfuric acid, mercury, chlorine, sulfur dioxide, and ethylene glycol [12, 13]. Reported laboratory measures of Radon concentration from national radon testing laboratories and state qualified radon testers or test kits [14, 15] were taken at specific sites and dates, typically triggered by inspection and report of violation of radon concentration regulation (i.e., <2 pCi/L) [16]. As such, these measures are likely not representative of the general exposure in a population. Considering that radon is a naturally occurring radioactive gas and the general exposure varies with the geological feature of an area, here we instead used the map of radon zones developed by the EPA [17] to represent the county-level radon exposure. Specifically, we regrouped the counties based on their radon zones as classified by the EPA [17]; counties in radon zones 1 and 2, where the predicted indoor radon levels exceed 2 pCi/L, were then labeled as high radon counties (reference group) and counties in radon zone 3 (predicted indoor radon levels below 2 pCi/L) were labeled as low radon counties.

2) Preventive healthcare coverage and general health conditions

Preventive healthcare coverage and general health conditions were another type of factors considered in this study. Several screenings were included, ranging from general health screenings (i.e., routine checkups and up-to-date preventative service) to specific cancer screenings (i.e., cervical cancer screening, breast cancer screening using mammography, and colorectal cancer screening using fecal occult blood test, sigmoidoscopy, and colonoscopy). All the health screening data were collected by the Behavior Risk Factor Surveillance System (BRFSS) in 2018 [18] and the county level estimates were generated by the PLACES Project [19], different from the 9-year averaged data we used for other risk factors. However, since data for other years were not available and we do not expect utilization of these preventive healthcare measures to change drastically over the 9 years, they were included in the analysis. Whenever a measure was taken for specific age and/or sex strata, we used the data for the relevant population strata alone (see details in Table S1). For instance, per cancer screening guidelines (e.g., women over 50 years old are recommended to take mammography [20]), the three cancer-screening related measures were age and sex specific (i.e., only applicable to specific age and sex); thus, these measures were only used for the relevant age- and sex-strata. In addition, mental and physical health status were assessed using two measurements collected by BRFSS and estimated by PLACES in 2018 [18, 19]; these are the percentage of people older than 18 years old with poor mental health over 14 days within a year and the percentage of people over 65 years old with all teeth lost.

3) Lifestyle factors

Unhealthy lifestyles are also well known for their adverse health effect. Here we assessed four representative unhealthy lifestyles, i.e., binge drinking, smoking, obesity, and lack of physical activities. Binge drinking data were obtained from BRFSS in 2018 and were not sex-specific. Data for the other three lifestyle risk factors were estimated by the Institute for Health Metrics and Evaluation (IHME) [21, 22] and were aggregated for each county from 2000-2009, for men and women, separately (Table S1).

4) Race composition and socioeconomic status (race & SES) measures

Three race & SES factors were included in our models based on well-documented cancer disparities by race/ethnicity and SES [23, 24]; these are related to race, poverty, and health insurance coverage. Specifically, race was measured by the percentage of white residents (including both Hispanic and non-Hispanic) in the county, the poverty level was measured by the percentage of people living in poverty, and health insurance coverage was measured by the percentage of people who lack health insurance. Data for all three factors were obtained from the American Community Survey (ACS) [25] and were averaged over 2010-2018 (i.e., without the time-lag).

Education is also an indicator of socioeconomic status. However, the percentage of people not completing high school education was highly correlated with the percentage of people living in poverty and the percentage of people who lack health insurance (Pearson’s correlation coefficient *r* = 0.70 and *r* = 0.73, respectively). As such, we did not include education in our models.

5) Spatial differences and community physical characteristics

For spatial differences, as noted in the main text, preliminary testing comparing spatial models (spatial autoregressive model (SAR), spatial Durbin model (SDM), and spatial error model (SEM)) and linear regression models did not find better model-fits using the spatial models (per likelihood ratio test). Thus, for simplicity, we used linear regression models and included the latitude of each county to account for any spatial differences among counties [26]. In addition, we included two measures on community physical characteristics; these are the percentage of agriculture land use derived by the Multiple-Resolution Land Characteristics Consortium (MRLC) from the National Land Cover Database (NLCD) [27], and the urban-rural classification based on a scheme developed by National Center of Health Statistics (NCHS) from NCHS data systems [28].

***Results of the sensitivity analyses***

Results from the three sets of sensitivity analyses are in general consistent with those from the main analysis. In the 1^st^ sensitivity analysis, when we relaxed the inclusion criterion, only one additional risk factor was identified in multiple models using the *P*<0.3 threshold, i.e., haloacetic acids concentration in drinking water (positive associations with thyroid cancer in men aged 50-69 and women aged 50-84; Table S5). In the 2^nd^ sensitivity analysis, when we used a more stringent correlation criterion to address multicollinearity, only 18 models were identified (vs. 22 in the main analysis); among them, 15 were the same as identified in the main analysis (Table S6). In the 3^rd^ sensitivity analysis, when we did not adjust for race (i.e., percentage of white residents in the county), 6 models for men and 7 models for women differed from those in the main analysis (Table S7). Of the 6 affected models for men, two did not meet the model goodness-of-fit criterion (i.e., R^2^ > 0.3) when race was not adjusted for. For the remaining 4 models, two found race significantly associated with cancer incidence (i.e., melanoma of the skin for men aged 25-49 and 70-84) and two (melanoma of the skin and thyroid cancer for men aged 50-59) identified risk factors likely connected with race (i.e., community physical characteristics and spatial pattern). Of the 7 affected models for women, four (i.e., breast cancer, corpus uteri cancer, kidney and renal pelvis cancer for women aged 25-49; thyroid cancer for women aged 70-84) found a significant association between race and cancer incidence. For the other 3 models, radon was additionally identified as a risk factor for melanoma of the skin for women aged 50-69 and thyroid cancer for women aged 70-84, and black carbon in PM_2.5_ was additionally identified as a risk factor for thyroid cancer for women aged 50-69.

**Reference:**

1. National Aeronautics and Space Administration (NASA) Goddard Space Flight Center. *NASA Ozone Watch: Ozone Facts*. Available from: <https://ozonewatch.gsfc.nasa.gov/facts/SH.html>.

2. The National Institute for Occupational Safety and Health (NIOSH). *NIOSH Pocket Guide to Chemical Harzards: Ozone*. Available from: <https://www.cdc.gov/niosh/npg/npgd0476.html>.

3. United States Environmental Protection Agency (EPA), *Air Quality System Data Mart [internet database]* <http://www.epa.gov/ttn/airs/aqsdatamart>.

4. United States Environmental Protection Agency (EPA), *RSIG-Related Downloadable Data Files*.

5. United States Environmental Protection Agency (EPA). *Nitrogen Dioxide (NO2) Pollution*. Available from: <https://www.epa.gov/no2-pollution>.

6. Cooper, M.J., et al., *Global fine-scale changes in ambient NO2 during COVID-19 lockdowns.* Nature, 2022. **601**(7893): p. 380-387.

7. Hammer, M.S., et al., *Global Estimates and Long-Term Trends of Fine Particulate Matter Concentrations (1998–2018).* Environmental Science & Technology, 2020. **54**(13): p. 7879-7890.

8. van Donkelaar, A., et al., *Regional Estimates of Chemical Composition of Fine Particulate Matter Using a Combined Geoscience-Statistical Method with Information from Satellites, Models, and Monitors.* Environmental Science & Technology, 2019. **53**(5): p. 2595-2611.

9. Hakim, M., et al., *Volatile organic compounds of lung cancer and possible biochemical pathways.* Chem Rev, 2012. **112**(11): p. 5949-66.

10. Philip, S., et al., *Spatially and seasonally resolved estimate of the ratio of organic mass to organic carbon.* Atmospheric Environment, 2014. **87**: p. 34-40.

11. National Environmental Public Health Tracking (EPH). *Indicator*. Available from: <https://ephtracking.cdc.gov/indicatorPages>.

12. Agency for Toxic Substances and Disease Registry (ATSDR), *National Toxic Substance Incidents Program (NTSIP) Annual Report*. 2010.

13. Agency for Toxic Substances and Disease Registry (ATSDR), *Hazardous Substances Emergency Events Surveillance data file*.

14. National Environmental Public Health Tracking (EPH). *Indicator: Radon Testing*. Available from: <https://ephtracking.cdc.gov/indicatorPages>.

15. National Environmental Public Health Tracking (EPH). *Indicator: Radon Tests from Labs*. Available from: <https://ephtracking.cdc.gov/indicatorPages>.

16. Centers for Disease Control and Prevention. *Test Your Home*. Available from: <https://www.cdc.gov/radon/radon-test.html>.

17. United States Environmental Protection Agency (EPA). *EPA map of Radon Zones*. Available from: <https://www.epa.gov/radon/epa-map-radon-zones>.

18. Behavioral Risk Factor Surveillance System (BRFSS), *2018 BRFSS Survey Data and Documentation*.

19. PLACES: Local Data for Better Health. Available from: <https://www.cdc.gov/places/index.html>.

20. U.S. Preventive Services Task Force. *Recommendation: Breast Cancer: Screening*. Available from: <https://www.uspreventiveservicestaskforce.org/uspstf/recommendation/breast-cancer-screening>.

21. Institute for Health Metrics and Evaluation (IHME), *United States Physical Activity and Obesity Prevalence by County 2001-2011.*

22. Institute for Health Metrics and Evaluation (IHME), *United States Smoking Prevalence by County 1996-2012.*

23. Zavala, V.A., et al., *Cancer health disparities in racial/ethnic minorities in the United States.* British journal of cancer, 2021. **124**(2): p. 315-332.

24. Ward, E., et al., *Cancer disparities by race/ethnicity and socioeconomic status.* CA: a cancer journal for clinicians, 2004. **54**(2): p. 78-93.

25. American Community Survey (ACS).

26. New York State GIS Program, *NYS Civil Boundaries*.

27. Multiple-Resolution Land Characteristics Consortium (MRLC), *National Land Cover Database*.

28. National Center for Health statistics (NCHS), *NCHS Urban-Rural Classification Scheme for Counties*.

## **Supplemental tables**

## Table S1. Risk factor data descriptions. Column “Type” shows the type of risk factor; column “Abbreviation” shows abbreviations of risk factor names; column “Measure” shows the specific measures; column “Year” shows the measurement years data were used in the analyses; column “Source” specifies the data sources. Column “Statistics” shows the mean (standard deviation) of each measure over the specified years; whenever available, sex-specific measures are shown and were used in the analyses.

| Type | | Abbreviation | Measure | Year | Source | Statistics | |
| --- | --- | --- | --- | --- | --- | --- | --- |
|  |  |  |  |  |  | Men | Women |
| Race & SES | | POV | Percent of population living in poverty | 2010-2018 | ACS [25] | 14.21 (3.75) | |
|  |  | INS | Percent of population without health insurance | 2010-2018 | ACS [25] | 8.68 (1.55) | |
|  |  | RACE | Percentage of white residents | 2010-2018 | ACS [25] | 0.9 (0.1) | 0.9 (0.12) |
| Env | Air Quality | OZONE | Ozone: Number of days with maximum 8-hour average concentration exceed NAAQS (monitor and modeled data) | 2000-2009 | EPA [3, 4] | 11.2 (4.72) | |
|  |  | NO2 | Annual mean ground-level NO2 mixing ratio for 2005-2019 | 2005-2009 | ACAG [6] | 1.71 (1.33) | |
|  |  | OMOC | Spatially and seasonally resolved estimate of the ratio of global organic mass to organic carbon | 2005-2008 | ACAG [10] | 2.29 (0) | |
|  |  | BC | PM_2.5_: Annual mean black carbon concentration (BC) | 2000-2009 | EPA [3],  ACAG [7, 8] | 0.8 (0.21) | |
|  |  | NH4 | PM_2.5_: Annual mean ammonium concentration (NH4+) | 2000-2009 | EPA [3],  ACAG [7, 8] | 1.48 (0.2) | |
|  |  | NIT | PM_2.5_: Annual mean nitrate concentration (NO3–) | 2000-2009 | EPA [3],  ACAG [7, 8] | 1.15 (0.28) | |
|  |  | OM | PM_2.5_: Annual mean organic matter concentration (OM) | 2000-2009 | EPA [3],  ACAG [7, 8] | 3.25 (0.39) | |
|  |  | SO4 | PM_2.5_: Annual mean sulfate concentration (SO42+) | 2000-2009 | EPA [3],  ACAG [7, 8] | 3.48 (0.42) | |
|  |  | SOIL | PM_2.5_: Annual mean mineral dust concentration (SOIL) | 2000-2009 | EPA [3],  ACAG [7, 8] | 0.36 (0.07) | |
|  |  | SS | PM_2.5_: Annual mean sea-salt concentration (SS) | 2000-2009 | EPA [3],  ACAG [7, 8] | 0.32 (0.13) | |
|  | Water Quality | HAA5 | Mean concentration of HAA5 (micrograms per liter) by year | 2000-2009 | EPH-state recipient [11] | 20.8 (12.62) | |
|  |  | TTHM | Mean concentration of TTHM (micrograms per liter) by year | 2000-2009 | EPH-state recipient [11] | 35.51 (14.18) | |
|  |  | NITRA | Mean concentration of Nitrate (milligrams per liter) by year | 2000-2009 | EPH-state recipient [11] | 0.85 (0.68) | |
|  | Other | TOXIC | Rate of reported acute toxic substance release incidents per 100,000 population | 2000-2009 | Hazardous Substances Emergency Events Surveillance (HSEES) [13] | 6.36 (3.14) | |
|  |  | RADONZONE | Radon zone | 2000-2009 | EPA [17] | 34 (54.84%) | |
| Health | | MH | Percent of adults aged >=18 years with mental health not good for >=14 days | 2018 | BRFSS [18], PLACES [19] | 13.22 (1.09) | |
|  |  | TL | Percent of adults aged >=65 years with all teeth loss | 2018 | BRFSS [18], PLACES [19] | 16.35 (2.67) | |
|  |  | CERVSCN | Percent of adult women aged >=21 to =65 years who have been screened for cervical cancer | 2018 | BRFSS [18], PLACES [19] |  | 85.09 (1.5) |
|  |  | CHKUP | Percent of adults aged >=18 years with visits to the doctor for routine checkup | 2018 | BRFSS [18], PLACES [19] | 80.75 (1.12) | |
|  |  | COLSIG | Percent of adults aged >=50 to =75 years who have received a fecal occult blood test, sigmoidoscopy, or colonoscopy. | 2018 | BRFSS [18], PLACES [19] | 67.19 (2.78) | |
|  |  | UTDPRV | Percent of older adult aged >=65 years who are up-to-date on a core set of clinical preventive services | 2018 | BRFSS [18], PLACES [19] | 25.22 (2.82) | 24.84 (2.12) |
|  |  | MAMMO | Percent of women aged >=50 to =74 years who use mammography | 2018 | BRFSS [18], PLACES [19] |  | 75.25 (2.41) |
| Lifestyle | | BINDRK | Percent of adults aged >=18 years who binge drink | 2018 | BRFSS [18], PLACES [19] | 17.46 (1.49) | |
|  |  | OBESE | Percent of adults aged >=18 years with obesity | 2000-2009 | IHME [21] | 31.88 (3.5) | 30.41 (3.06) |
|  |  | PHYACT | Percent of adults aged >=18 years with no leisure-time physical activity | 2000-2009 | IHME [21] | 25.89 (2.96) | 22.7 (2.75) |
|  |  | SMOK | Percent of adults aged >=18 years with current smoking | 2000-2009 | IHME [22] | 23.62 (3.21) | 25.76 (2.69) |
| Other | | AG | Percent of land used for agriculture | 2000-2009 | NLCD [27] | 23.19 (17.45) | |
|  |  | RURAL | Classification of county from rural to urban (two category scale) | 2000-2009 | NCHS [28] | 36 (58.06%) | |
|  |  | LAT | Latitude of county | 2000-2009 | NYS GIS Program [26] | 42.56 (1) | |
|  |  | LAT2 | Polynomial term of latitude | 2000-2009 | NYS GIS Program [26] | 1812.07 (84.74) | |

## Table S2. Estimated associations for 25–49-year-olds. The top row shows cancer type, for which satisfactory models were identified (adjusted R^2^ > 0.3). The 2^nd^ row shows sex (men and women). Each column shows results for each cancer type, sex, and model; colored cells show variables included in the model (see variable names in the ‘Measure’ column), i.e., white cells indicate the corresponding variables are not included. All variables were standardized to 0 mean and 1 standard deviation (SD); thus, the numbers (mean and 95% confidence intervals in parentheses) show estimated number of SD increase in cancer incidence rate for each SD increase in the exposure. Colors indicate the direction of the association (blue = negative; red = positive). Asterisks (*) indicate statistically significant at a = 0.05 level.


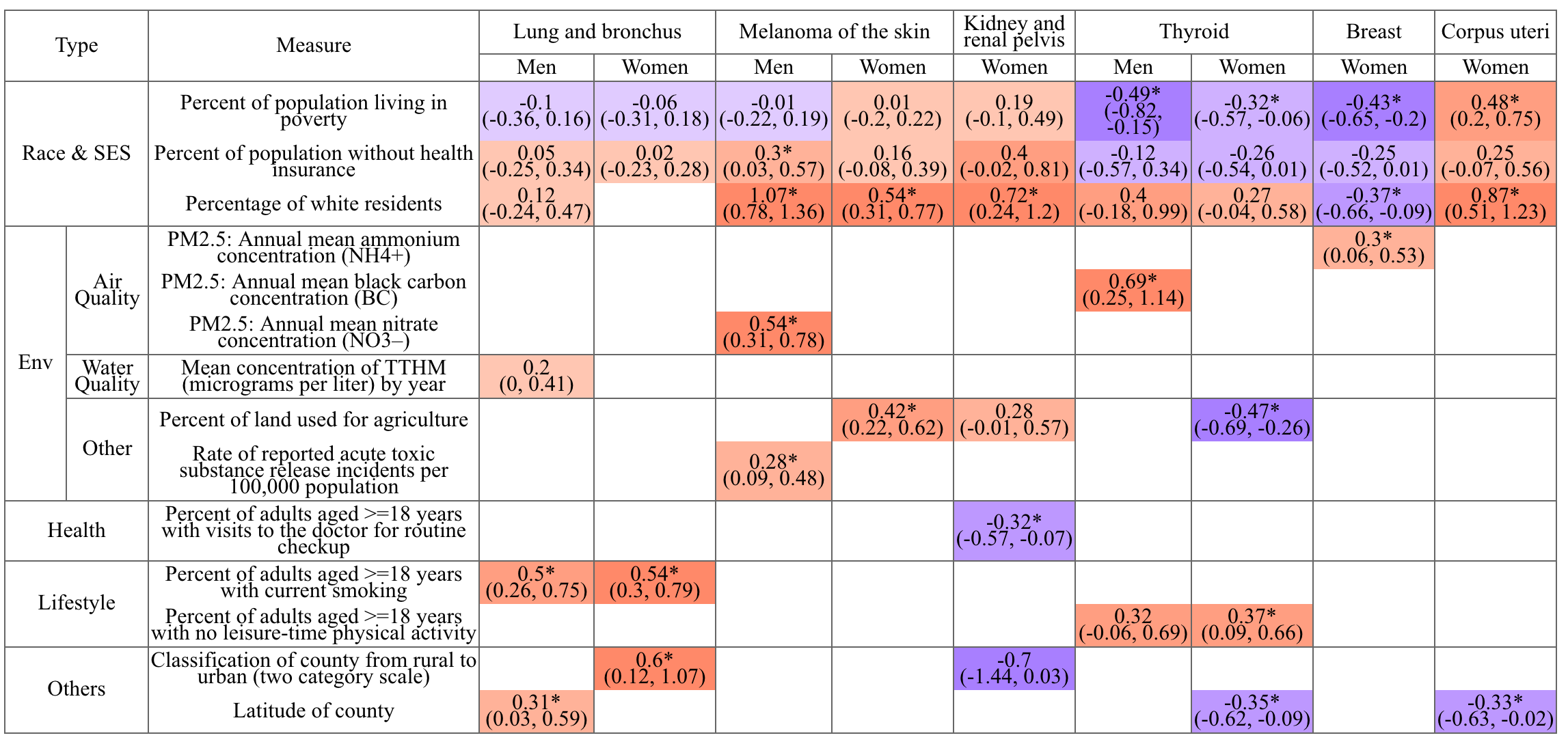


## Table S3. Estimated associations for 50–69-year-olds. The top row shows cancer type, for which satisfactory models were identified (adjusted R^2^ > 0.3). The 2^nd^ row shows sex (men and women). Each column shows results for each cancer type, sex, and model; colored cells show variables included in the model (see variable names in the ‘Measure’ column), i.e., white cells indicate the corresponding variables are not included. All variables were standardized to 0 mean and 1 standard deviation (SD); thus, the numbers (mean and 95% confidence intervals in parentheses) show estimated number of SD increase in cancer incidence rate for each SD increase in the exposure. Colors indicate the direction of the association (blue = negative; red = positive). Asterisks (*) indicate statistically significant at a = 0.05 level.


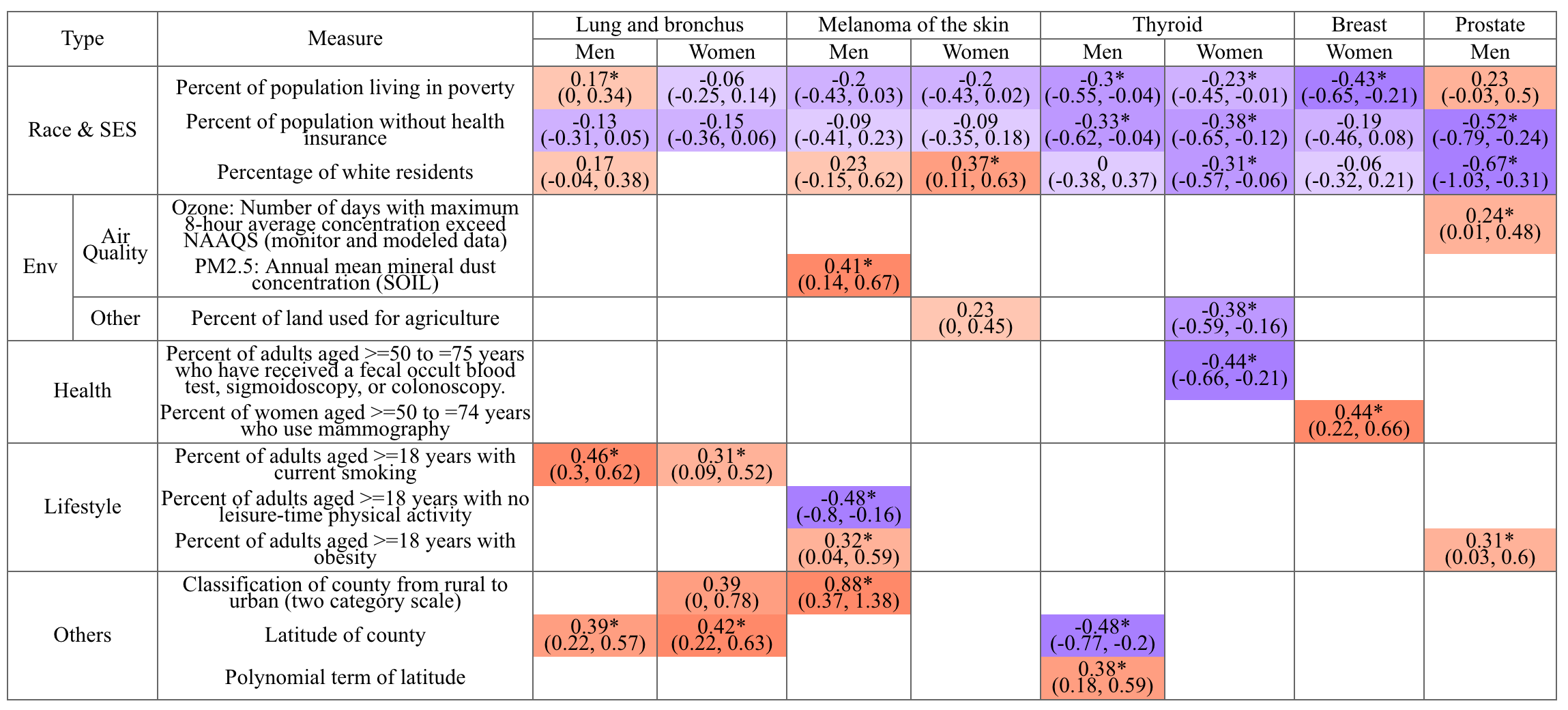


## Table S4. Estimated associations for 70–84-year-olds. The top row shows cancer type, for which satisfactory models were identified (adjusted R^2^ > 0.3). The 2^nd^ row shows sex (men and women). Each column shows results for each cancer type, sex, and model; colored cells show variables included in the model (see variable names in the ‘Measure’ column), i.e., white cells indicate the corresponding variables are not included. All variables were standardized to 0 mean and 1 standard deviation (SD); thus, the numbers (mean and 95% confidence intervals in parentheses) show estimated number of SD increase in cancer incidence rate for each SD increase in the exposure. Colors indicate the direction of the association (blue = negative; red = positive). Asterisks (*) indicate statistically significant at a = 0.05 level.


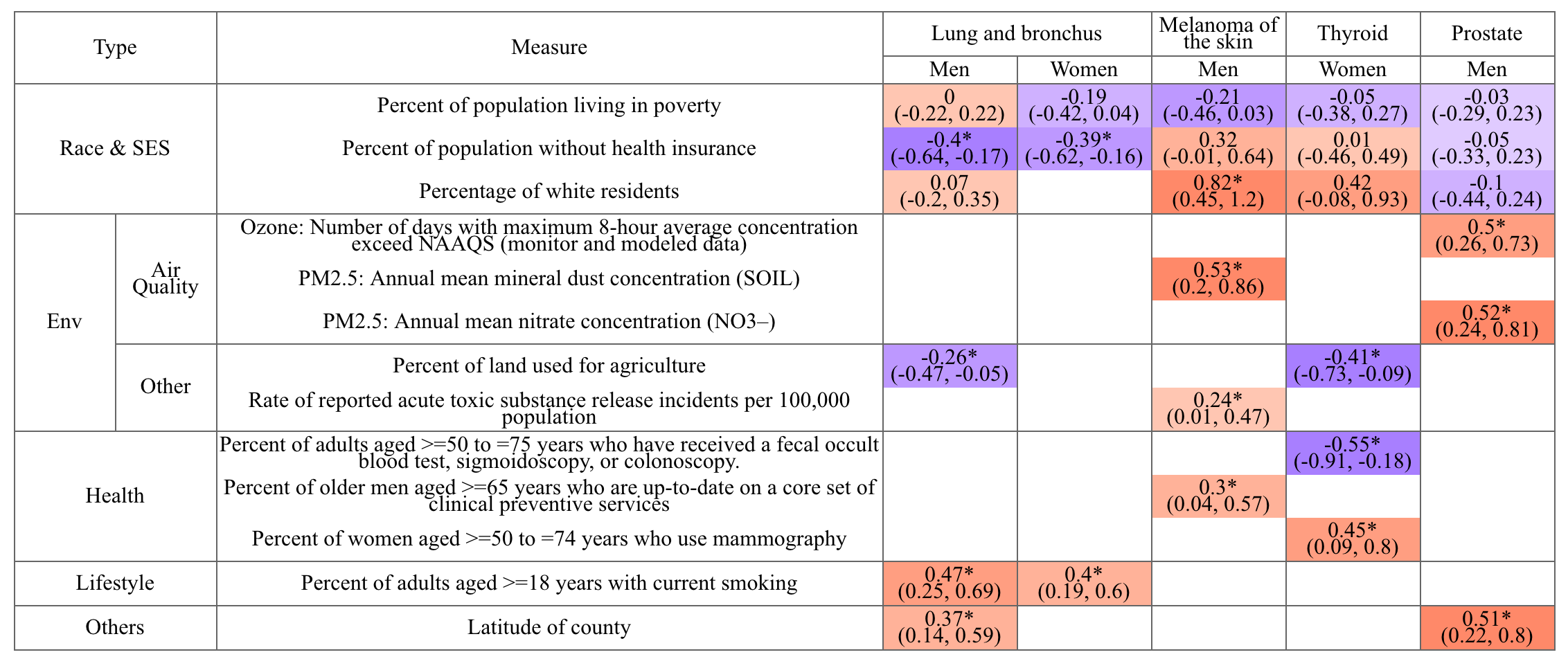


## Table S5. Sensitivity analysis on the threshold P-value used in the individual risk factor analysis to pre-select risk factors for the full multivariate analysis. There were 22 models with satisfactory performance (i.e., adjusted R^2^ >0.3) in total and only those with non-identical results are shown here (i.e., the ones with consistent results across p-value settings are not shown). Each row shows results for each model (for a given cancer, sex, and age group, as indicated by the first three columns). Columns 4-7 show the risk factors identified by the best model when the threshold p-value was set to 0.1, 0.2, and 0.3, respectively; alternative models with similar performance are also presented in parentheses.

| Cancer | Sex | Age group | P <0.1  (Main analysis) | P <0.2 | P <0.3 |
| --- | --- | --- | --- | --- | --- |
| Lung and bronchus | Women | 25-49 | SMOK, SS  (SMOK, RURAL) | Same as 0.3 | HAA5, SMOK, RURAL, LAT  (SMOK, SS; SMOK, RURAL) |
| Kidney and renal pelvis | Women | 25-49 | RURAL, AG, CHKUP | Same as 0.1 | AG, CHKUP, OM, PHYACT |
| Melanoma of the skin | Women | 50-69 | AG | Same as 0.1 | OZONE, RADONZONE, LAT |
| Thyroid | Men | 50-69 | LAT, LAT2 | Same as 0.3 | HAA5, LAT, LAT2 |
| Thyroid | Women | 50-69 | AG, COLSIG | Same as 0.1 | HAA5, LAT, COLSIG, AG, OZONE |
| Thyroid | Women | 70-84 | MAMMO, COLSIG, AG | BINDRK, MAMMO, COLSIG, AG | BINDRK, MAMMO, COLSIG, AG, HAA5 |

## Table S6. Sensitivity analysis on the correlation threshold used in the full multivariate analysis. In the analysis, any variable pair with a Pearson’s correlation above the set threshold was excluded in the same model to reduce multicollinearity issues. There were 22 models with satisfactory performance (i.e., adjusted R^2^ >0.3) when the correlation threshold was set to 0.6 and 18 models when the threshold was set to 0.5. For clarity, only those with non-identical results are shown here. Each row shows results for each model (for a given cancer, sex, and age group, as indicated by the first three columns). Columns 4 and 5 show the risk factors identified by the best model when the correlation threshold was set to 0.5 and 0.6, respectively.

| Age group | Cancer | Sex | Correlation threshold=0.5 | Correlation threshold=0.6 (Main analysis) |
| --- | --- | --- | --- | --- |
| 25-49 | Lung and bronchus | Women | No models with R^2^ > .3 identified | INS, POV, SMOK, LAT |
| 25-49 | Thyroid | Men | No models with R^2^ > .3 identified | INS, POV, RACE, PHYACT, AG, LAT |
| 25-69 | Kidney and renal pelvis | Women | No models with R^2^ > .3 identified | INS, POV, RACE, RURAL, CHKUP, AG |
| 50-69 | Lung and bronchus | Women | INS, POV, LAT | INS, POV, SMOK, LAT, RURAL |
| 50-69 | Melanoma of the skin | Men | INS, POV, RACE, OZONE, AG | INS, POV, RACE, RURAL, PHYACT, OBESE, SOIL |
| 50-69 | Prostate | Men | INS, POV, RACE, OZONE | INS, POV, RACE, OBESE, OZONE |
| 70-84 | Lung and bronchus | Women | INS, POV, RACE | INS, POV, SMOK |
| 70-84 | Thyroid | Women | No models with R^2^ > .3 identified | INS, POV, RACE, MAMMO, COLSIG, AG |

Table S7. Sensitivity analysis on model adjustment for race. Each row shows results for each model (for a given cancer, sex, and age group, as indicated by the first three columns). Column 4 shows the risk factors identified using models adjusting for race as in the main analysis; Column 5 shows the risk factors identified using models without adjusting for race. Alternative models with similar performance are also presented in parentheses. Note the three models for lung and bronchus for women were not included in the table, because in the main analysis those models did not adjust for race due to high correlations with smoking.

| Sex | Age group | Cancer | Adjusting for race  (main analysis) | Without adjusting for race (sensitivity analysis) |
| --- | --- | --- | --- | --- |
| Men | 25-49 | Lung and bronchus | RACE, SMOK, TTHM, LAT | (SAME but no RACE) |
|  |  | Melanoma of the skin | RACE, TOXIC, NO_3_ | AG, RACE |
|  |  | Thyroid | RACE, PHYACT, BC | No models with R^2^ > .3 identified |
|  | 50-69 | Lung and bronchus | RACE, SMOK, LAT | (SAME but no RACE) |
|  |  | Melanoma of the skin | RACE, PHYACT, OBESE, SOIL | AG, RURAL, HAA5 |
|  |  | Prostate | RACE, OZONE, OBESE | No models with R^2^ > .3 identified |
|  |  | Thyroid | RACE, LAT, LAT2 | HAA5, LAT, LAT2 |
|  | 70-84 | Lung and bronchus | RACE, SMOK, LAT, AG | (SAME but no RACE) |
|  |  | Melanoma of the skin | RACE, SOIL, TOXIC, UTDPRV | AG, RACE |
|  |  | Prostate | RACE, OZONE, LAT, NIT | (SAME but no RACE) |
| Women | 25-49 | Breast | RACE, NH4 | (SAME) |
|  |  |  | (RACE, SO4) | LAT, OM |
|  |  | Corpus uteri | RACE, TTHM | (SAME) |
|  |  |  | (RACE, LAT) | SMOK, OM |
|  |  | Kidney and renal pelvis | RACE, CHKUP, RURAL, AG | RACE, CHKUP, AG |
|  |  | Melanoma of the skin | RACE, AG | SAME |
|  |  | Thyroid | RACE, AG, PHYACT, LAT | AG, PHYACT |
|  | 50-69 | Breast | RACE, MAMMO | SAME |
|  |  | Melanoma of the skin | RACE, AG | RADONZONE, LAT, NIT, OM |
|  |  | Thyroid | RACE, AG, COLSIG | AG, COLSIG, BC |
|  | 70-84 | Thyroid | RACE, AG, COLSIG, MAMMO | (SAME) |
|  |  |  |  | AG, COLSIG, BC, RADONZONE |

## Table S8. Correlations between obesity and race & SES variables in model analyses of female breast and colorectal cancer. Note that some counties had missing data or reported low cancer incidence rates and thus were not included in the analysis; thus, the corrections were computed using data specific for each cancer, age group, and sex.

| Cancer | Sex | Age | POV | INS | RACE |
| --- | --- | --- | --- | --- | --- |
| Breast | Women | 25-49 | 0.61 | 0.17 | 0.57 |
|  |  | 50-69 |  | 0.14 | 0.56 |
|  |  | 70-84 |  |  |  |
| Colon and rectum | Men | 25-49 | 0.43 | 0.13 | 0.50 |
|  |  | 50-69 |  |  |  |
|  |  | 70-84 |  |  |  |
|  | Women | 25-49 | 0.61 | 0.14 | 0.57 |
|  |  | 50-69 |  | 0.17 |  |
|  |  | 70-84 |  | 0.14 | 0.56 |

Fig S1. Analysis Flowchart. Given the large number of study factors (31 in total) and small number of counties (62 or less depending on availability of data), we first used bivariate and race & SES-adjusted models to examine individual risk factors and identify the ones showing likely association with cancer risk. The variable subset from the individual risk factor analysis was then further examined in a full multivariate analysis (steps in the double-border box). See main text for detail.

##
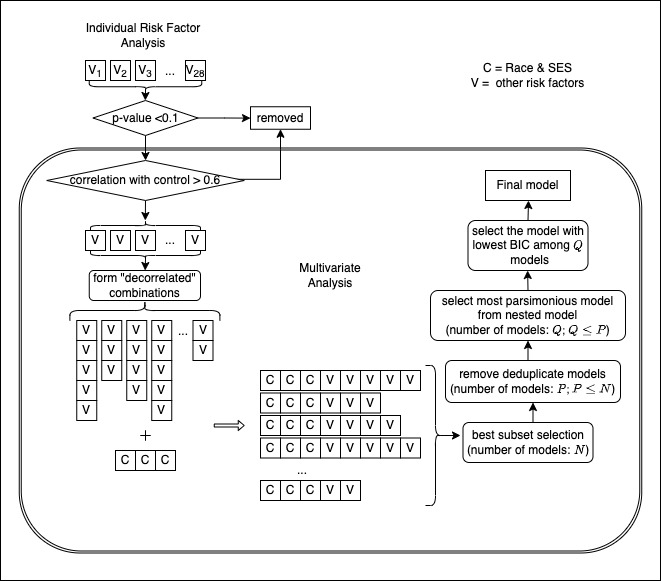


## Fig S2. Risk factors included in the study for each cancer, sex, and age group. Each row represents a type of cancer for each age group and sex. Risk factors in grey were not included in either the individual risk factor analysis or the multivariate analysis (those in lighter grey were excluded because they are highly correlated with the race & SES variables; those in darker grey were not available for/relevant to the corresponding age group and sex). Those in yellow are race & SES variables and included in both analyses. Those in orange were included in the individual risk factor analysis and were not selected in the subsequent multivariate analysis. Those in red were included in both analyses (i.e. selected after the individual risk factor analysis). Those marked with an “X” were selected in the final best-model. See main text for detail on model selection.


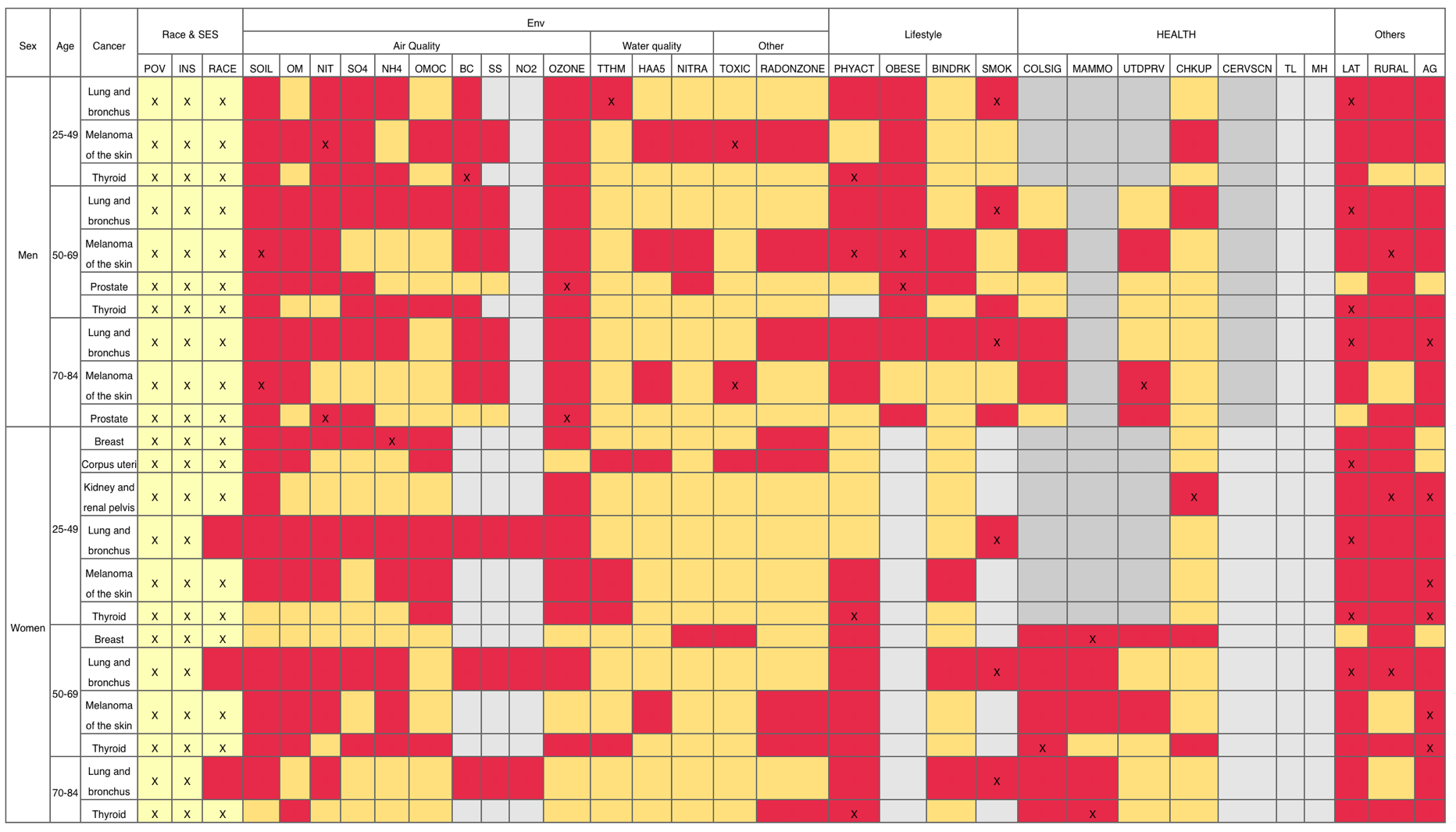


Fig S3. Pairwise correlations of environmental variables examined for men. Each dot shows the Pearson’s correlation (*r*) for the corresponding variable pair (see x- and y-axis labels and full variable names in Table S1); the dot size and color (see legend) indicate the magnitude (also shown by the numerical value). Black boxes denote variable pairs with *r* > 0.6 and were thus not included in the same model.


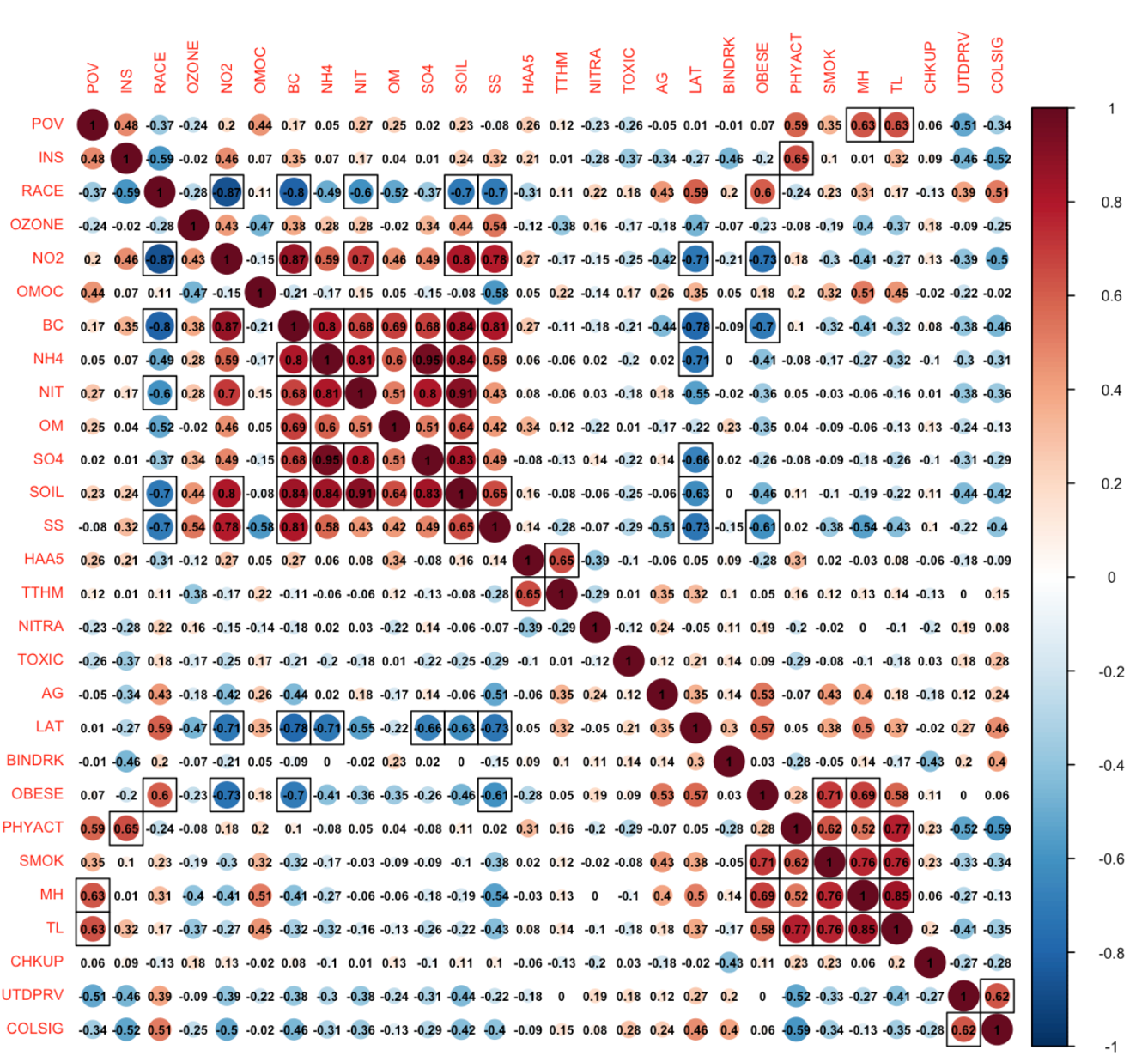


Fig S4. Pairwise correlations of environmental variables examined for women. Each dot shows the Pearson’s correlation (*r*) for the corresponding variable pair (see x- and y-axis labels and full variable names in Table S1); the dot size and color (see legend) indicate the magnitude (also shown by the numerical value). Black boxes denote variable pairs with *r* > 0.6 and were thus not included in the same model. Note that some variables (e.g., MAMMO = mammography) are sex-specific.


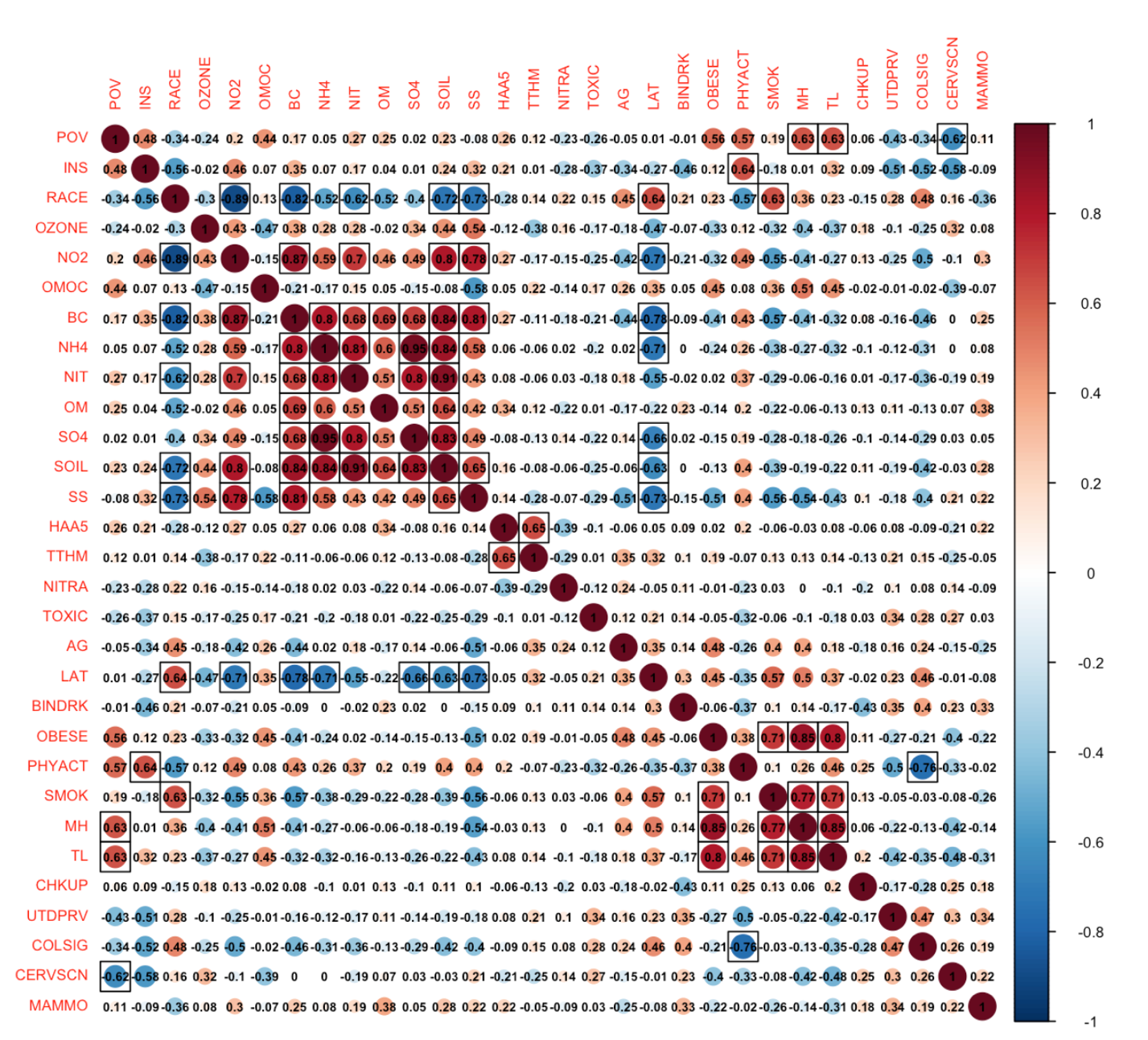


## Fig S5. Spatial pattern of thyroid cancer and lung and bronchus cancer incidence. Models identified associations with county latitude (a proxy measure for spatial differences) for these two cancers (see Table 3 for estimates).


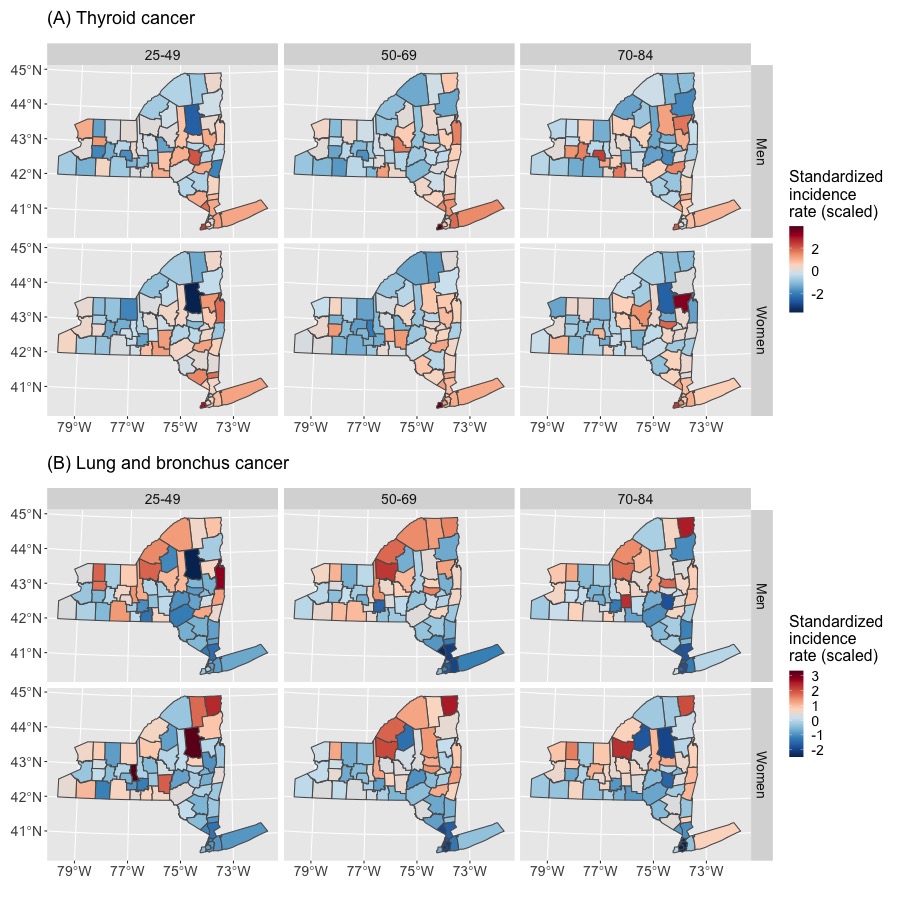

Supplement: Supplementary file 1 — Supplementary Information. [file 41598_2024_56634_MOESM1_ESM.docx]
